# Genome-wide characterization of the β-1,3-glucanase gene family in *Gossypium* by comparative analysis

**DOI:** 10.1038/srep29044

**Published:** 2016-06-29

**Authors:** Xiaoyang Xu, Yue Feng, Shuai Fang, Jun Xu, Xinyu Wang, Wangzhen Guo

**Affiliations:** 1 State Key Laboratory of Crop Genetics & Germplasm Enhancement, Hybrid Cotton R&D Engineering Research Center, MOE, Nanjing Agricultural University, Nanjing 210095, China; 2 College of Life Science, Nanjing Agricultural University, Nanjing 210095, China

## Abstract

The β-1,3-glucanase gene family is involved in a wide range of plant developmental processes as well as pathogen defense mechanisms. Comprehensive analyses of β-1,3-glucanase genes (*GLUs*) have not been reported in cotton. Here, we identified 67, 68, 130 and 158 *GLUs* in four sequenced cotton species, *G. raimondii* (D_5_), *G. arboreum* (A_2_), *G. hirsutum* acc. TM-1 (AD_1_), and *G. barbadense* acc. 3–79 (AD_2_), respectively. Cotton *GLUs* can be classified into the eight subfamilies (A–H), and their protein domain architecture and intron/exon structure are relatively conserved within each subfamily. Sixty-seven *GLUs* in *G. raimondii* were anchored onto 13 chromosomes, with 27 genes involved in segmental duplications, and 13 in tandem duplications. Expression patterns showed highly developmental and spatial regulation of *GLUs* in TM-1. In particular, the expression of individual member of *GLUs* in subfamily E was limited to roots, leaves, floral organs or fibers. Members of subfamily E also showed more protein evolution and subgenome expression bias compared with members of other subfamilies. We clarified that *GLU42* and *GLU43* in subfamily E were preferentially expressed in root and leaf tissues and significantly upregulated after *Verticillium dahliae* inoculation. Silencing of *GLU42* and *GLU43* significantly increased the susceptibility of cotton to *V. dahliae.*

The hydrolysis of 1,3-β-D-glucosidic linkages in β-1,3-glucans is catalyzed by the enzymes of β-1,3-glucanases (E.C. 3.2.1.39), which are widely found in bacteria, fungi, viruses^1^,^2^ and various plant species, including *Arabidopsis*^3^, rice^4^, tobacco^5^ and soybean^6^. β-1,3-Glucanase genes (*GLUs*) have formed complex and diverse gene families in plants, where they play important roles in a wide range of physiological and developmental processes^3^. In plants, the interest in β-1,3-glucanase has focused primarily on their antifungal activity. For example, plant β-1,3-glucanases, classified as the PR-2 family of pathogenesis-related proteins, have been proposed to defend against pathogen infection in two ways: by hydrolyzing β-1,3-glucan, a major structural component of fungal cell walls; and by promoting the release of cell wall associated immune elicitors that further stimulate defense reactions^7^,^8^. In other areas, there is strong evidence that these glycosyl hydrolases have also played the important roles in pollen development^4^,^9^, seed germination^10^, cold response^11^ and regulation of plasmodesmata signaling^12^,^13^.

According to the diversity in its C-terminal domain, plant β-1,3-glucanase has been divided into different types, and each type contains an N-terminal signal peptide and a glycoside hydrolase family 17 domain^3^. In tobacco, β-1,3-glucanases are grouped into five classes^5^,^14^. Class I comprises basic proteins and exhibits developmental regulation. These proteins contain a short C-terminal signal sequence that is believed to be the responsible for vacuolar localization. In contrast to class I, the class II and III proteins are acidic proteins that are induced by pathogens. They appear to be localized in the extracellular spaces because they lack the C-terminal signal sequence. The class IV and V proteins are also acidic proteins with no C-terminal extension, however, they have different expression pattern when compare to class II and III proteins. The class IV proteins, including sp41a and sp41b, are expressed in the style of tobacco flowers and are not pathogen inducible. Tag1 belongs to class V and is expressed exclusively in the tobacco anther tapetum. In *Arabidopsis*, β-1,3-glucanases are also classified into three major clades (denoted α, β and γ) based on their evolutionary relationships^3^.

Cotton is one of the most important economic crops worldwide, and provides the world’s leading natural textile fiber and considerable amounts of edible oil. Among approximately 50 species in the *Gossypium* genus, only four, two diploids (*G. arboreum* and *G. herbaceum*) and two allotetraploids (*G. hirsutum* and *G. barbadense*), have been independently domesticated for the production of economically valuable fibers. Previous studies have shown that allotetraploid *Gossypium* species were formed by a polyploidization event that occurred 1–2 million years (Myr) ago, which involved a maternal A-genome species resembling *G. arboreum* and a paternal D-genome species resembling *G. raimondii*^15^. With its high yield properties, *G. hirsutum* (commonly called Upland cotton) contributes over 90% of the annual global commercial cotton production. Nevertheless, cotton production is limited by diverse biotic and abiotic stresses. *Verticillium* wilt is a widespread disease that occurs in a range of cotton producing areas. More than 50% of the cotton acreage is influenced by *Verticillium* wilt in some years, significantly reducing the fiber quality and yield (National Cotton Council, 2012 http://www.cotton.org/). β-1,3-Glucanases have been proposed to play important roles in physiological and developmental processes, as well as in the response of plants to microbial pathogens, and show great functional diversity between the members of this large gene family^7^. In order to obtain an integrated image of the evolutionary characteristics and possible functions of the β-1,3-glucanase gene family in *Gossypium*, it is necessary to develop genome-wide analyses of the gene family at various levels. The publically available genomic information from four sequenced cotton species, *G. raimondii*^16^, *G. arboreum*^17^, *G. hirsutum*^18^ and *G. barbadense*^19^, provides us with a data source to identify candidate *GLUs* at a genome-wide level in *Gossypium*, and to mine key genes for the genetic improvement of yield and fiber quality, as well as disease resistance.

In this study, we individually identified *GLUs* from four sequenced cotton species, and analyzed their chromosomal location, sequence phylogeny, genomic structure and expansion pattern. We carried out a genome-wide analysis of the temporal and spatial expression profiles of *GLUs* in *G. hirsutum*, and investigated their molecular evolution and subgenome expression divergence. Further, specific focus was placed on the *GLUs* in subfamily E and virus-induced gene silencing (VIGS) analysis confirmed that silencing of the two *GLUs* in subfamily E significantly increased the susceptibility of cotton to *V. dahlia*. Our study provides a basis for systematically elucidating the evolutionary and functional characteristics of *GLUs* in cotton, for the effective clarification of the precise biological roles of *GLUs* and their utilization in future cotton-breeding programs.

## Results

### Genome-wide identification of the β-1,3-glucanase gene family in *Gossypium*

**Figure 1:**
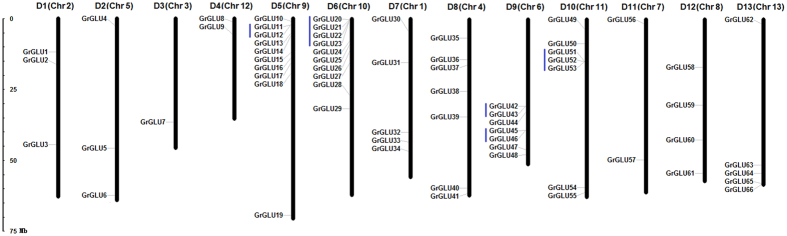
Chromosomal distribution of β-1,3-glucanase genes in *G. raimondii*. The chromosome numbers were consistent with the interspecific genetic map (D1 to D13) in allotetraploid cultivated cotton species^20^ and the scaffolds (Chr.1 to Chr.13) in the genomic data of *G. raimondii*^16^. The nomenclature of *GLUs* was based on the order of the chromosomes in *G. raimondii*. Lines were drawn to indicate the tandem duplicated genes.

The whole genome sequence scaffolds of four sequenced cotton species (*G. raimondii*, *G. arboreum*, *G. hirsutum* acc. TM-1 and *G. barbadense* acc. 3–79) were used for the genome-wide exploration of the β-1,3-glucanase gene family in *Gossypium*. With data from a query on the glycoside hydrolase family 17 domain (PF00332), we searched the protein databases of four cotton species using HMMER v3.0 software. In total, 67 *GLUs* in *G. raimondii* were obtained after confirming the “Glyco_hydro_17” domain with a blastp program and these were named *GrGLU1* through *GrGLU*67 by combining their chromosome order (D1 to D13)^20^ with their location on the chromosome (Fig. 1). The chromosomal distribution patterns of these *GrGLUs* were uneven. Chrs D5 and D6 contained the most *GLUs* (10 genes), while Chr D3 contained the fewest (one gene). Further, we identified 68 *GLUs* in *G. arboreum*. As these had an orthologous relationship to the *GLUs* in *G. raimondii*, the 67 *GLUs* in *G. arboreum* were named *GaGLU1-67*, corresponding to *GrGLU1-67*. Notably, *GaGLU54* had undergone a tandem duplication event in *G. arboreum*, and the two tandem duplication genes were named *GaGLU54a* and *GaGLU54b*.

From a phylogenetic point of view, there were two homologous genes (homeologs from the A and D subgenomes) in the tetraploid cotton species. In total, we identified 130 *GLUs* in *G. hirsutum* acc. TM-1 (named *GhGLU1-67*A/D; Supplementary Fig. 1) and 158 in *G. barbadense* acc. 3–79, and these candidate *GLUs* in *G. barbadense* experienced more tandem duplications at the subgenome level. More than 90% of the *GLUs* had homoeologous genes in the two tetraploid cotton species, indicating the independent evolution of the A- and D-subgenomes after polyploid formation. Other inconsistencies might result from different sequencing methods, assembly error in partial chromosomal regions, or different degrees of colonization during the evolutionary process of *Gossypium*, and need to be further confirmed. In *G. hirsutum* acc. TM-1, the chromosomal location of *GhGLUs* in the D subgenome shows good collinearity with that in the D genome (*G. raimondii*), however, two reciprocal translocations between the A2 (Chr2) and A3 (Chr3) and between the A4 (Chr4) and A5 (Chr5) chromosomes were detected in *G. hirsutum*^18^ (Supplementary Fig. 1). Information on the *GLUs* in two diploid and two allotetraploid cotton species is summarized in Supplementary Table 1.

### Classification and structural analysis of β-1,3-glucanases

**Figure 2:**
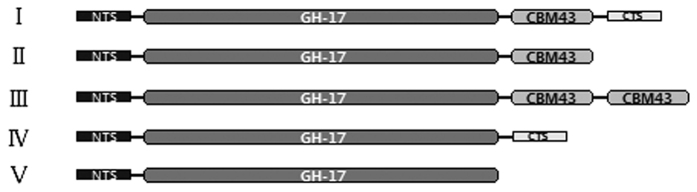
Protein domain architectures of β-1,3-glucanases in cotton and *A. thaliana*. NTS: N-terminal sequence. GH-17: glycosyl hydrolase family 17 domain. CBM43: carbohydrate-binding modules family 43. CTS: hydrophobic C-terminal sequence. These five architectural types are based on the presence/absence of CMB43 and CTS in C-terminal.

Each ortholog from the four surveyed cotton species had a similar protein structure. With the information on the *GrGLUs* in *G. raimondii* as an example of the further analysis carried out, all 67 *GrGLUs* contained an N-terminal signal peptide and a glycoside hydrolase family 17 domain. In detail, five types of protein domain architectures (type I to type V) were observed in the *GrGLUs* (Fig. 2), which was consistent with that in *Arabidopsis*^3^. The carbohydrate-binding modules family 43 (CBM43) domain, which has the ability to bind β-1,3-glucan^21^, was found in 39 of the 67 *GrGLUs*. A C-terminal hydrophobic sequence encoding a transient transmembrane domain was present in 42 *GrGLUs*. The transient transmembrane domain may be a vacuolar targeting peptide^22^ or a glycosylphosphatidylinisotol (GPI)-anchor attachment^23^,^24^. Further, we compared the number of *GrGLUs* with that in *A. thaliana*, *Theobroma cacao* and *Vitis vinifera* for each protein domain architectural type (Table 1). Type III was the smallest, with only one member in *G. raimondii* and *A. thaliana*, and none in *T. cacao* or *V. vinifera*. Type I was the largest, with 23 members in *G. raimondii*, 19 in *A. thaliana* and 16 in *T*. *cacao*. It is reasonable to suggest that the ancestral *GLUs* in plants may have the protein domain architecture as type I, with a CBM43 domain and a C-terminal hydrophobic sequence after the core glycosyl hydrolase family 17 domain, and these genes may play an important role in cell division or cell wall remodeling as they have abundant expression in a variety of tissues and organs. During the evolutionary process, the loss of the C-terminal region, including the GPI anchor, and the alteration of expression patterns of duplicated genes by acquiring or losing of regulatory *cis*-elements, led to the formation of new *GLUs*, which were classified as types II to V, and resulting in their functional diversity^3^.

### Phylogenetic analysis of β-1,3-glucanase genes

**Figure 3:**
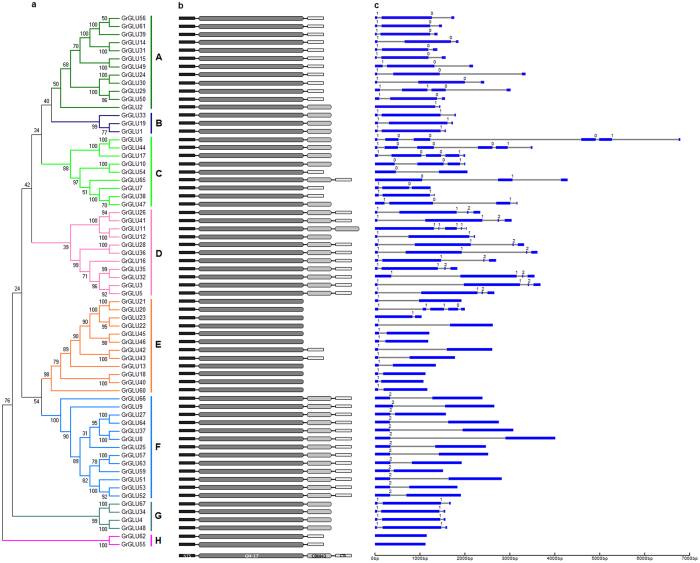
Phylogenetic relationships, protein domain architecture and gene structures of β-1,3-glucanases in cotton. **(a)** The multiple alignment of the conserved glycosyl hydrolase family 17 domain were constructed with Clustal X (version 2.0)^49^, and gaps and poorly aligned sections were removed (Supplementary Fig. 3). Phylogenetic tree was generated using the maximum likelihood method under WAG model in MEGA v5.2^50^, and the reliability of interior branches was assessed with 1000 bootstrap resamplings. **(b)** Protein domain architectures as defined in Fig. 2. (**c**) The gene structures were drawn using the online tool GSDS. Introns and exons were represented by black lines and blue boxes, respectively, and numbers at the exon-intron joints were intron phases.

Systematic classifications of *GLUs* at a genome-wide level have been reported previously in *Arabidopsis*^3^ and tobacco^5^. To gain further insights into the evolutionary relationships, we employed MEGA v5.2 software to construct an unrooted phylogenetic tree of *GLUs* from *G. raimondii*, *A. thaliana* and tobacco. The phylogenetic tree clearly showed that the 123 *GLUs* could be clustered into eight subfamilies (A–H) (Supplementary Fig. 2), with *At5g67469* and *GrGLU58* (which contain a partial “Glyco_hydro_17” domain) as outgroup. All five classes of tobacco *GLUs* fall into subfamily E. Arabidopsis clades β and γ were consistent with subfamilies E and F, respectively, and the *GLUs* in clade α fall into the other subfamilies. The *GrGLUs* were classified into eight subfamilies, and this phylogenetic classification was consistent with the protein domain architecture and exon-intron organization (Fig. 3). Subfamilies A, C, D, E and F each contained more *GrGLUs*, with the number of members ranging from 9 to 13, while subfamilies B, G and H each contained only 3, 4 and 2 *GrGLUs*, respectively.

The CBM43 domain and the hydrophobic C-terminal sequence were found to be subfamily dependent. All members of subfamilies B, D, F and G contained the CBM43 domain, whereas it was absent from the members of subfamily E. The C-terminal hydrophobic sequence was present in members of subfamilies A, F and H, but was absent from members of subfamilies B and G. With the exception of subfamily C, and some members of subfamily D, such as *GrGLU11* and *GrGLU12*, and subfamily E, such as *GrGLU20* and *GrGLU23*, the introns in most of the *GrGLUs* were highly subfamily-specific (Fig. 3), indicating that *GLUs* in different subfamilies may have undergone independent gene duplication in *G. raimondii*.

### β-1,3-Glucanase gene family expansion in the *Gossypium* genus

Previous studies have suggested that there was at least one additional whole genome duplication (WGD) after the pan-eudicot triplication in the *Gossypium* genome, however, the frequency and timing of the event are still being debated^16^,^25^. To investigate the β-1,3-glucanase gene family expansion pattern in cotton, we download the syntenic data of *G. raimondii*, *A. thaliana*, *V. vinifera* and *T. cacao* from the Plant Genome Duplication Database (PGDD). *A. thaliana* has undergone at least three WGD events, including two doublings (α and β events) since its divergence from other members of the Brassicales clade^26^ and one tripling (γ event) which was shared with most if not all eudicots^27^. The *V. vinifera* genome has experienced no WGD since the pan-eudicot triplication, and it is regarded as an excellent reference for determining the numbers of WGDs in eudicots^28^. Of the whole genome sequenced plants, *T. cacao* is most closely related to cotton and had no WGD after the ancient hexaploidization event^16^,^29^.

**Figure 4:**
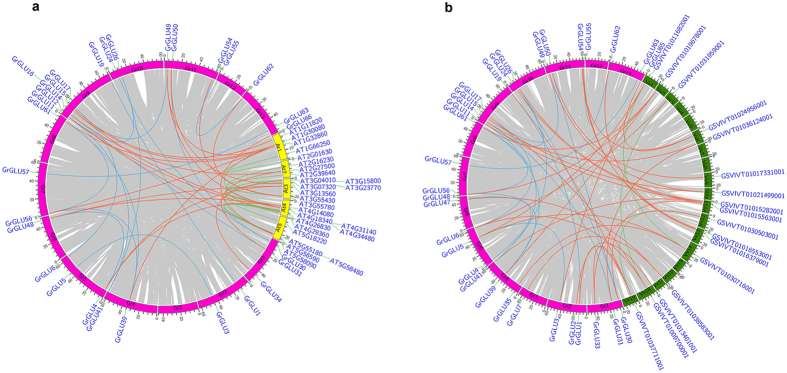
The intra- and inter-genomic comparison showed gene synteny of β-1,3-glucanase genes in *G. raimondii* (Gr), *A. thaliana* (At) and *V. vinifera* (Vv). The gray lines indicated whole genome duplication blocks. The light blue and green lines indicated the intra-genomic synteny of β-1,3-glucanase genes in *G. raimondii*, *A. thaliana* **(a)** and *V. vinifera* **(b)**, and the orange lines indicated the inter-genomic synteny of β-1,3-glucanase genes between species.

The WGD and tandem duplication of *GLUs* were analyzed to elucidate the gene family expansion events in *G. raimondii*. Among the 67 *GrGLUs* in *G. raimondii*, we identified 27 genes within 17 pairs of syntenic blocks (Fig. 4; Supplementary Table 2). The *Ks* values for each pair of genes within a syntenic block were used to interpret duplication events (Table 2). For two pairs of paralog genes, *GrGLU6* vs *GrGLU17* and *GrGLU30* vs *GrGLU50*, the *Ks* values were 2.260 and 2.034, respectively, and these two paralog gene pairs may be derived from the ancient hexaploidization event. For the remaining 15 paralog gene pairs, the *Ks* values ranged from 0.436 to 0.806, implying that these paralog gene pairs originated from the *Gossypium* lineage WGD events^16^,^25^. We also analyzed the adjacent genes to investigate whether tandem duplication had taken place. 13 of the 67 *GrGLUs* showed tandem duplication, with a cluster of 4 members (*GrGLU20*, *GrGLU21*, *GrGLU22* and *GrGLU23*) present in Chr D6 (Fig. 1).

Furthermore, we analyzed the gene duplication pattern of *GLUs* from different phylogenetic subfamilies. Inter-genomic synteny analysis showed that *GLUs* with syntenic relationships were detected in all subfamilies with the exception of subfamily E, and this indicated that the gene orders of the blocks in subfamily E were less conserved during the evolution of diploid cotton. In total there were 12 pairs of genes with syntenic relationships in subfamilies A, B, C, G and H, but these were not generated by tandem duplication events. In subfamily E, which contained 12 *GLUs*, there were no genes with syntenic relationships but 8 tandem duplicated genes were present. In subfamilies D and F, there were 7 genes within different syntenic blocks and 5 tandem duplicated genes (Figs 1 and 4; Table 2). These results show that *GLUs* from different phylogenetic subfamilies have different expansion patterns. In summary, *Gossypium* lineage WGD event contributed to the expansion of subfamilies A, B, C, G and H, while subfamily E expanded through tandem duplication events. WGD and tandem duplication worked together to promote subfamily D and F expansion.

**Figure 5:**
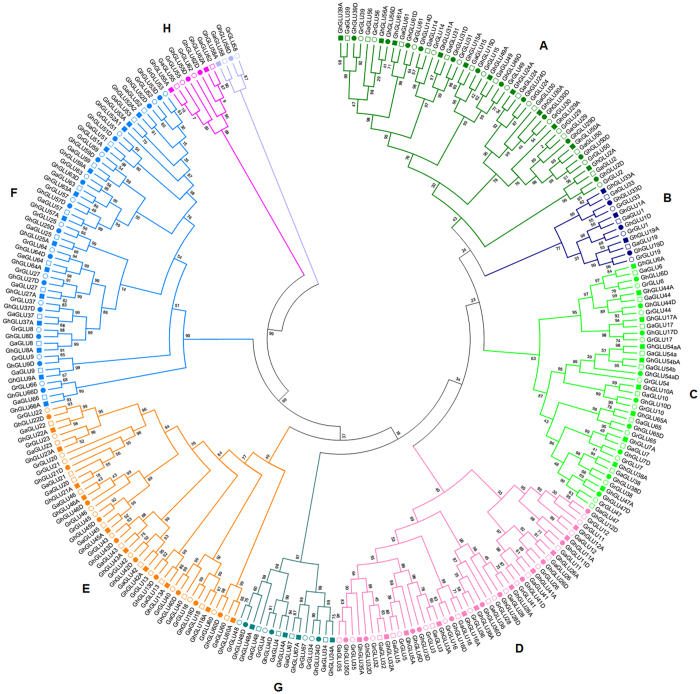
Phylogenetic relationships of β-1,3-glucanase genes in diploid and allotetraploid cotton. Amino acid sequences of conserved glycosyl hydrolase family 17 domain were aligned using ClustalX, and gaps and poorly aligned sections were removed. Phylogenetic tree was generated using the maximum likelihood method under WAG model in MEGA v5.2, and the reliability of interior branches was assessed with 1000 bootstrap resamplings.

It has been suggested that allotetraploid cotton species appeared in the last 1–2 Myr through hybridization and subsequent polyploidization events that involved a maternal A-genome species and a paternal D-genome species. As a relatively young polyploidy species, genes from parental genomes were mostly preserved in the *G. hirsutum* subgenomes^18^. Here, we identified 66 and 64 *GLUs* in the A and D subgenomes of *G. hirsutum* acc. TM-1, respectively (Fig. 5; Supplementary Table 1). The number of *GLUs* in allotetraploid cotton has nearly doubled when compared with that in diploid cotton, although a handful of homologs have been lost.

### Expression patterns of β-1,3-glucanase genes in *G. hirsutum* acc. TM-1

**Figure 6:**
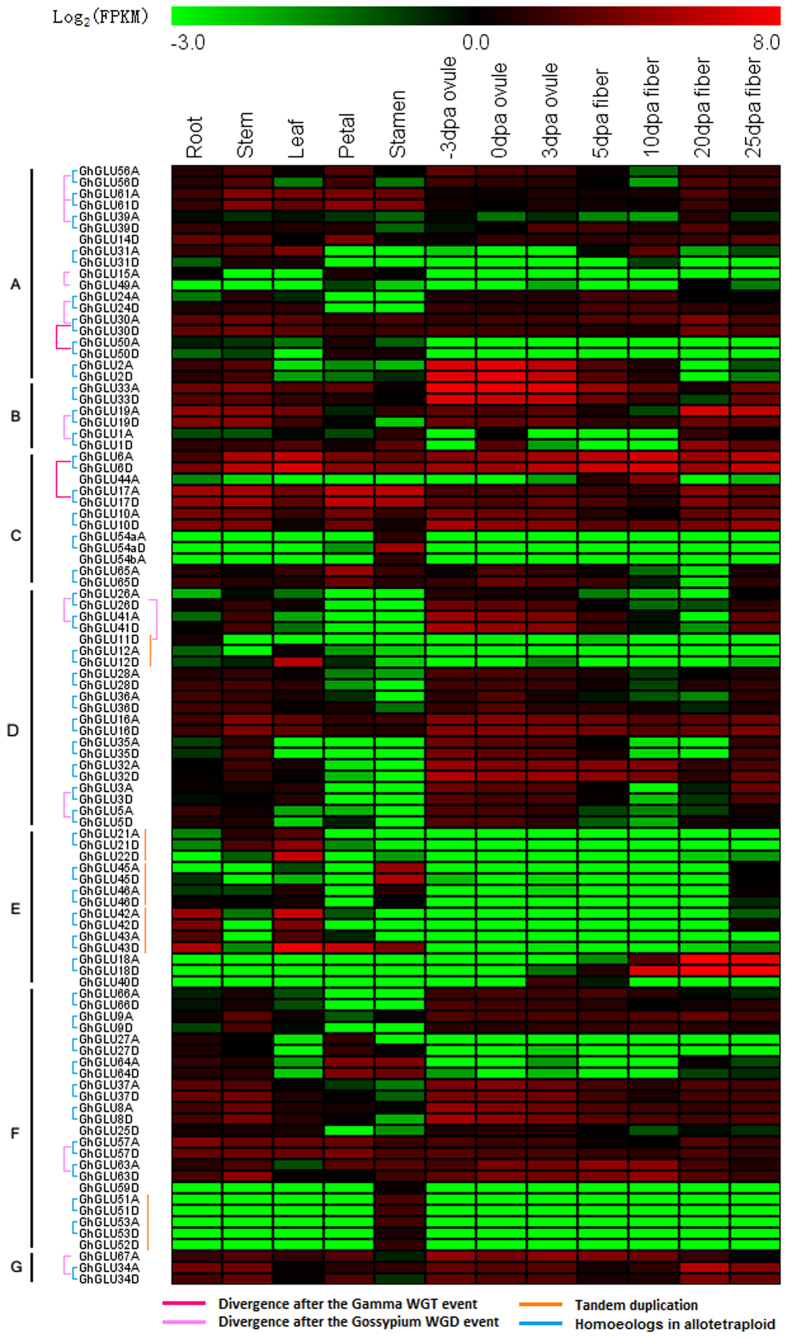
Expression profiles and their evolutional pattern of β-1,3-glucanase genes in cotton. The RNA-seq relative expression data of 12 tissues was used to re-construct expression patterns of β-1,3-glucanase genes in *G. hirsutum* acc. TM-1. The lines showed the blocks containing the corresponding β-1,3-glucanase genes experienced the WGD events, or tandem duplication events.

Previous studies have suggested that *GLUs* are expressed in all plant tissues and are regulated temporally and spatially depending on environmental conditions and developmental stage^3^,^7^. Recently-published research^18^, including *G. hirsutum* acc. TM-1 gene expression profiles, allowed us to investigate the expression of the β-1,3-glucanase gene family in different organs and developmental stages. The FPKM method was employed to normalize the total short read sequences. Among the 130 *GhGLUs*, there were 98 genes with an FPKM > 1 in at least one of the 12 investigated organs and developmental stages, and these 98 genes were used to gauge the relative expression of each β-1,3-glucanase gene (Fig. 6). The remaining 32 *GhGLUs* may be pseudogenes, or are only expressed at specific developmental stages, or under special environmental conditions that were not analyzed in this study. We observed that the accumulation of the 98 *GhGLUs* transcripts was associated with different organs and developmental stages, and that the expression patterns differed among each subfamily (Fig. 6). Notably, the expression of the individual member of *GLUs* in subfamily E was limited to roots, leaves, floral organs or cotton fibers, not all tissues we analyzed.

**Figure 7:**
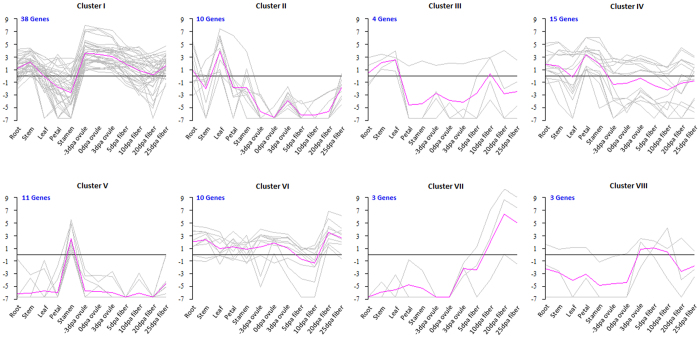
Expression clusters of β-1,3-glucanase genes in *G. hirsutum*. The gray line shows expression profile of each gene, and the purple line is the average expression of one cluster.

A hierarchical clustering analysis of expression profiles in the 12 tissues produced 8 clusters (Cluster I–VIII) comprising a total of 94 *GhGLU*s, and the remaining 4 genes could not be divided into any of the above clusters (Fig. 7, Supplementary Table 3). Thirty-eight *GhGLU*s were highly expressed in −3, 0 and 3 days post anthesis (DPA) ovules and fell into Cluster I. Members in this cluster also had high expression in stems but low expression in stamens. There were 14 *GhGLU*s in Clusters II and III and these were highly expressed in leaves. The *GhGLU*s in Cluster III were also highly expressed in stems. The expression of 26 genes was highest in petals or stamens and these fell into Clusters IV and V, respectively. The remaining 16 genes were highly expressed in the fibers and these fell into Clusters VI, VII and VIII. Expression of the *GhGLUs* in Clusters VI and VII was highest in the 20 DPA fibers, although transcripts of the genes in Cluster VI were also detected in vegetative and reproductive organs.

**Figure 8:**
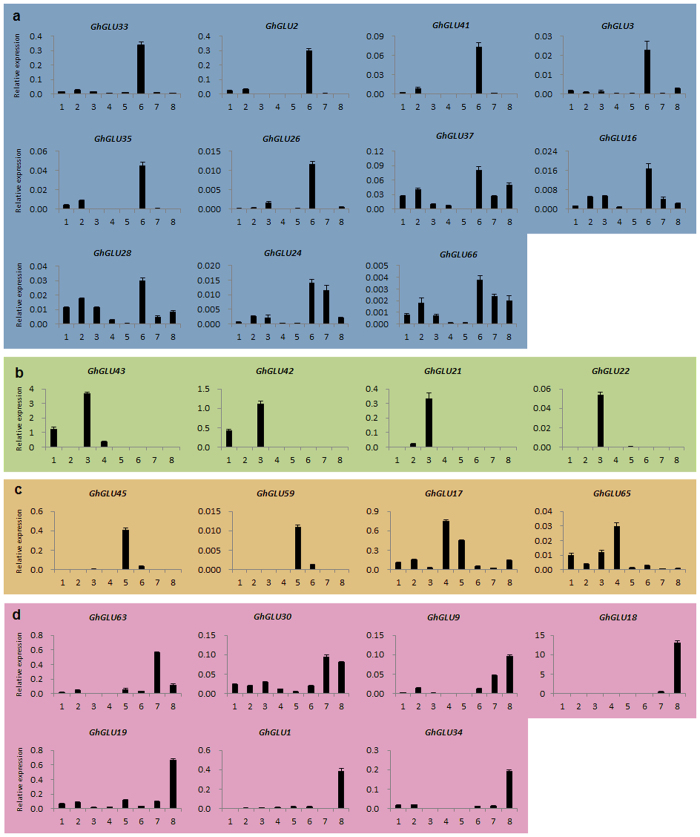
Expression patterns of *GhGLU*s in different tissues and developmental stages by qRT-PCR. 1: root; 2: stem; 3: leaf; 4: petal; 5: anther; 6: ovule at 0 DPA; 7: fiber at 10 DPA; 8: fiber at 20 DPA. **(a)** High expression in the ovule at 0 DPA **(b**) Expression peaks in the leaf **(c**) High expression in the flowers (**d**) High expression in the fibers.

To confirm the expression profiles above, qRT-PCR was conducted for 26 genes in 8 organs and developmental stages of TM-1 (Fig. 8). The qRT-PCR primers used (Supplementary Table 3) were gene-specific but not homoeolog-specific. Firstly, 11 *GhGLU*s showed expression peaks in 0 DPA ovules, and these were divided into two groups. *GhGLU33*, *GhGLU3*, *GhGLU41*, *GhGLU3*, *GhGLU35* and *GhGLU26* were predominantly found to accumulate in ovules at 0 DPA ovules, and *GhGLU37*, *GhGLU16*, *GhGLU24*, *GhGLU28* and *GhGLU66* were also detected in vegetative organs and fibers, with low expression levels in petals and anthers. Secondly, four *GhGLUs*, *GhGLU42*, *GhGLU43*, *GhGLU21* and *GhGLU22*, were predominantly found to accumulate in roots and leaves. Thirdly, the expression peaks of four *GhGLUs*, *GhGLU45*, *GhGLU59*, *GhGLU17* and *GhGLU65*, occurred mainly in petals or anthers. Lastly, seven *GhGLUs* were found to accumulate at high levels in fibers at 10 and 20 DPA. *GhGLU63* showed preferential expression in fibers at 10 DPA, and *GhGLU30* had similar expression levels in 10 and 20 DPA fibers, while *GhGLU9*, *GhGLU18*, *GhGLU19*, *GhGLU1* and *GhGLU34* were highly expressed in 20 DPA fibers (Fig. 8). The results of our qRT-PCR analysis of *GhGLUs* are consistent with the RNA-Seq data.

### Molecular evolution and subgenome expression divergence of β-1,3-glucanase genes

The whole genome sequencing of *G. raimondii*, *G. arboreum*, *G. hirsutum*, and *G. barbadense* has provided us with an opportunity to explore the molecular evolutionary properties of *GLUs* in diploids and their allotetraploid derivatives. We calculated the nonsynonymous substitution rate (*Ka*), synonymous substitution rate (*Ks*) and *Ka/Ks* ratios within and between species^30^ to explore the divergence and selection pressures that have accumulated in *GLUs* (Table 3). Average *Ka* and *Ks* values were higher in inter-genomic contrasts (A vs. D or At vs. Dt) than in intra-genomic contrasts (A vs. At or D vs. Dt), and this is consistent with the hypothesis that the A–D genome divergence occurred well before the formation of the allotetraploid species. To our surprise, the average *Ka* of *GLUs* in subfamily E was higher than that in other subfamilies, indicating that members of subfamily E experienced quicker protein evolution. The overall low *Ka/Ks* ratios in the inter- and intra-genomic contrasts suggested negative or purifying selection of *GLUs* in both diploid and polyploid cotton species, however, subfamily E had a higher *Ka/Ks* ratio in At vs. Dt than other subfamilies, with A vs. D as the control. Therefore, *GLUs* in subfamily E experienced more protein evolution in both diploid and polyploid cotton, and they also underwent more selection pressures when compared to other subfamilies.

**Figure 9:**
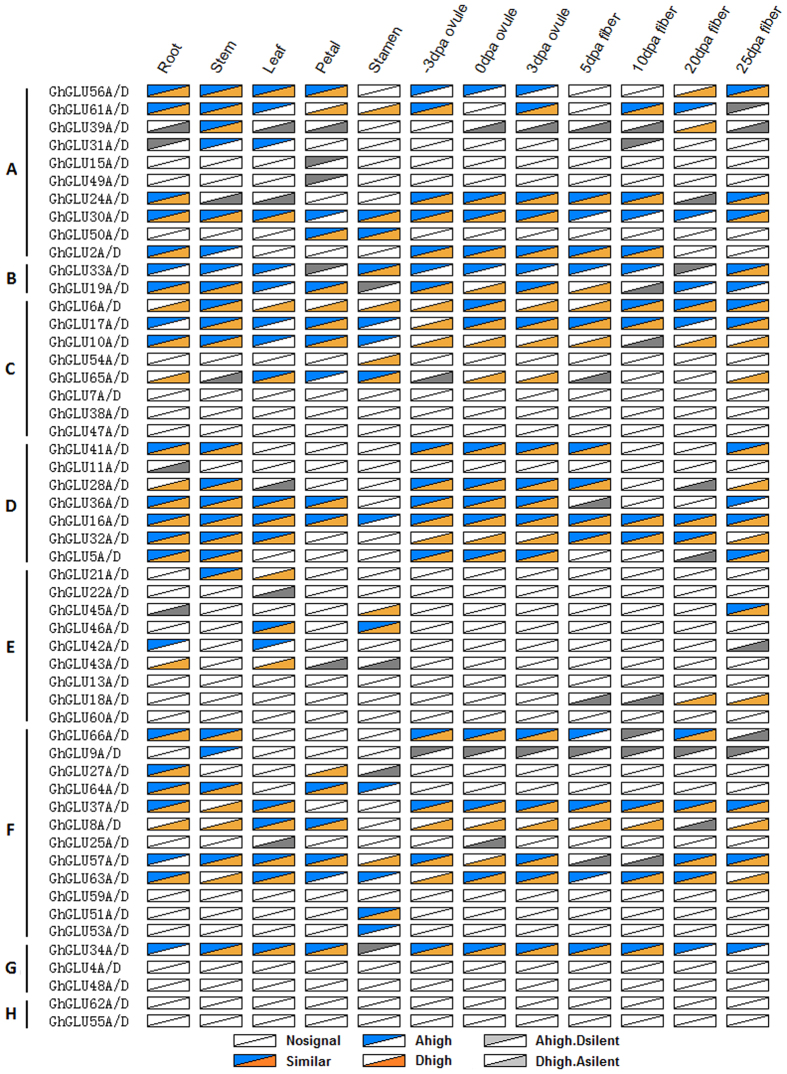
Ratios of β-1,3-glucanase homoeologs transripts in *G. hirsutum*. Nosignal: FPKM of both homoeologs was less than one. Similar: No expression divergence detected between homoeologs. Ahigh: A homoeolog expression was higher than D. Dhigh: D homoeolog expression was higher than A. Ahigh.Dsilent: A homoeolog expression was higher than D, and FPKM of D homoeolog was less than one. Dhigh.Asilent: D homoeolog expression were higher than A, and FPKM of A homoeologs was less than one. The higher expression level of homoelogs were detected through pairwise t-test (P value < 0.01, FDR < 0.05 and at least 1.5-fold difference in expression levels).

Through the genome-wide analysis of *GLU* expression levels in 12 TM-1 (*G. hirsutum*) tissues (53 homoeologs × 12 tissues = 636 combinations), we found that 147 (23.1%) homoeologous gene pairs showed similar expression levels, 340 (53.5%) pairs were undetectable, and 149 (23.4%) pairs were subgenome-biased (Fig. 9). Of the 149 subgenome-biased pairs, 52 pairs showed one gene expressed while the other was undetectable, 45 pairs showed higher levels in A than D subgenome (A subgenome bias), and 52 pairs showed higher levels in D than A subgenome (D subgenome bias). In particular, only 19 homoeologous gene pairs in subfamily E were found to have transcripts in the tested tissues, of them, there were 15 (78.9%) pairs showed subgenome bias expression, implying specific roles for members in subfamily E during the evolutionary process.

### Potential function of two tandem duplicated β-1,3-glucanase genes in *Verticillium dahliae* resistance

**Figure 10:**
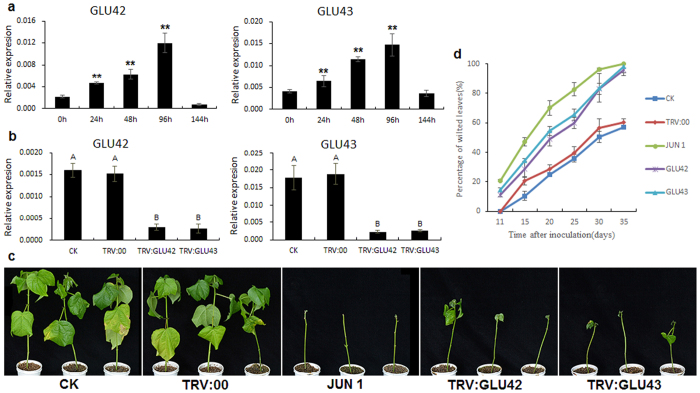
Silencing of *GLU42* and *GLU43* enhances cotton susceptibility to *Verticillium dahlia* infection. **(a**) Expression analysis of *GLU42* and *GLU43* after inoculation by *V. dahliae*. Asterisks indicate significant expression differences compared inoculated time with 0 h (“*”: t test at P < 0.05, “**”: t test at P < 0.01). **(b)** Expression of *GLU42* and *GLU43* in silenced and control plant leaves by qRT-PCR analysis. Different letters indicate the significant expression difference each other (Tukey’s multiple comparison test, P < 0.01). **(c)** Plant phenotypes at 30 days after *V. dahlia* inoculation. **(d**) Percentage of diseased leaves after *V. dahlia* inoculation. The experiments were repeated using 20 plants per treatment. The error bars were calculated based on three independent experiments using standard deviation. Percentage of diseased leaves for each treatment was showed in Supplementary Table 4. Different letters indicate the significant difference each other (Tukey’s multiple comparison test, P < 0.05).

Four tandem duplicated *GLUs* from subfamily E, *GLU42* and *GLU43*, and *GLU21* and *GLU22*, were found to be predominantly accumulated in leaves, and *GLU42* and *GLU43* were also detected in roots (Fig. 8). qRT-PCR showed that *GLU42* and *GLU43* were significantly induced after *Verticillium dahliae* inoculation, with the highest expression observed at 96 h post-inoculation (Fig. 10a).

To investigate the function of *GLU42* and *GLU43* in *Verticillium* resistance, we constructed TRV2:*GLU42* and TRV2:*GLU43* vectors to silence the endogenous genes in Hai7124, with TRV:00 as the mock treatment and TRV2:*GhCLA1* (Cloroplastos alterados 1) as an indicator. An Agrobacterium culture containing all recombinant pTRV vectors was infiltrated into the cotyledons of cotton cultivar Hai7124. Three weeks later, the plants infiltrated with *CLA1* exhibited the photobleaching phenotype (Supplementary Fig. 4a), and *GLU42* and *GLU43* were silenced in TRV2:*GLU42*- and TRV2:*GLU43*-infiltrated plants when compared to no-infiltration control (CK) and mock treated plants (Fig. 10b). Further, we found that *GLU42* was silenced in TRV2:*GLU43*-silenced plants, and *GLU43* was also silenced in TRV2:*GLU42* silenced plants. This may be due to the 90.98% nucleotide identity between the two tandem duplicated genes (Supplementary Fig. 5). We also performed qRT-PCR to examine the expression of other *GLUs* in subfamily E. Only two *GLUs*, *GLU21* and *GLU22*, were detected to express in leaf tissue, however their expression had not been interfered in *GLU42/43* silenced plants (Supplementary Fig. 6). After the *GLU42/43* were silenced, we inoculated the cotton seedlings by dip-infection with *V. dahliae* isolate V991, with the final concentration adjusted to 1 × 10^7^ spores per milliliter. Eleven days after inoculation, yellow leaf veins and wilting were found in cotyledons of *GLU42/43*-silenced plants. Similar phenotypes were observed in susceptible plants (*G. hirsutum* cv. Junmian 1), but not in no-infiltration control and mock treated plants. Twenty days later, the true leaves of *GLU42/43*-silenced plants became withered and etiolated, and thirty days later, most of the true leaves were defoliated (Fig. 10c). In general, no-infiltration control and mock treated plants exhibited a weak partial leaf wilting phenotype. However, silencing of *GLU42/43* enhanced the plants’ susceptibility to *V. dahlia*: over 83% of *GLU42/43* silenced plant leaves exhibited severe wilting symptoms inoculated thirty days later, it’s higher than that of mock treated plants. Further, more than 96% *GLU42/43* silenced plant inoculated thirty-five days later were severely infected, similar to the results observed in susceptible control plants (*G. hirsutum* cv. Junmian 1) (Fig. 10c,d, Supplementary Table 4). No-inoculation control with silenced *GLU42/43* showed the normal growth and development (Supplementary Fig. 4b). The VIGS results indicated that *GLU42* and *GLU43* play important roles in resistance to *V. dahliae* infection in cotton, and have a potential utilization in cotton disease-resistant breeding.

## Discussion

Whole-genome duplication, followed by massive silencing and elimination of duplicated genes, has long been recognized as a pervasive force in plant evolution^31^. Complete genome analyses support the idea that two polyploidy events (ρ and σ) occurred in monocots, and one triplication event (γ) was probably shared by all core eudicots^26^,^27^,^32^. The *Gossypium* lineage underwent a WGD after the γ event, although the frequency and timing of this event are still being debated^16^,^25^. Meanwhile, the fate of the duplicated genes derived from WGD events is still poorly understood, both evolutionarily and functionally. Several studies have shown that some duplicate genes were more prone to retention than others. For example, transcription factors, signal proteins, and membrane proteins were preferentially retained after duplication in *Arabidopsis*^33^. The duplicate genes retained after an ancestral WGD event enhanced root nodule symbiosis in the Papilionoideae^34^.

In this study, we identified 67 *GrGLUs* in *G. raimondii*. Of them, 27 *GrGLUs* were detected within 17 pairs of syntenic blocks (Fig. 4; Table 2), with two paralogs derived from the ancient hexaploidization, and 15 paralogs from the *Gossypium* lineage WGD events. These paralogs were retained after the WGD events. We also detected 21 *GrGLUs* that showed there was only one β-1,3-glucanase gene remaining in the syntenic blocks (Supplementary Table 2), and further, their corresponding orthologs were also found in *A. thaliana* (Fig. 4a), *V. vinifera* (Fig. 4b) or *T. cacao* (Supplementary Table 2). This indicated the duplicated genes in the syntenic blocks were lost in *Gossypium* after WGD event. The retained paralogs might have gained new functions (neofunctionalization), lost functions (pseudogenization), or partitioned aggregate ancestral function (subfunctionalization)^35^,^36^. In *G. raimondii*, 99.4% of the duplicated genes were reported to exhibit differential expression in at least one of three tissues (petal, leaf, and seed)^37^. In this study, we also found that *GhGLU41* and *GhGLU26* showed different expression to *GhGLU11*, indicating the functional diversity of these paralogs. However, evolutionary mechanisms underlying the preservation and diversification of duplicated genes followed by WGD remain largely unknown.

According to parsimony reconstruction, the ancestral *GLUs* most probably displayed a somewhat nonspecific expression pattern and were involved in cell division or cell wall remodeling-like functions^3^. After several rounds of ancient WGD events in seed plants and angiosperms, the *GLUs* with ancestral functions may have diverged in expression; leading to the production of new functions in pathogen resistance, pollen development and tube growth, seed germination, and other processes^3^,^31^.

The sequence-based phylogeny of *GLUs* is not entirely consistent with the expression divergence in *Arabidopsis*^3^ and cotton in our study, possibly because duplicated genes acquired or lost tissue or developmental cis-regulatory elements during the evolutionary process. However, the *GLUs* in subfamily E were specifically expressed in particular tissues or organs. For example, the expression of *GhGLU18* (Accession number: D88416.1) specific to fibers at 20 DPA played an important role in reopening plasmodesmata by degrading callose during cotton fiber development^38^. An *Arabidopsis* β-1,3-glucanase gene, *AtBG_pap*, is considered as a plasmodesmal gate keeper for intercellular communication^12^,^13^. Due to the temporary deposition of callose, a transient closure of plasmodesmata occurs during the rapid phase of cotton fiber elongation. The expression of *GhGLU18* highly correlates with the timing of plasmodesmata opening, and could play a role in cotton fiber development by degrading callose in plasmodesmata^38^,^39^,^40^. The expression of *GhGLU21*, *GhGLU22*, *GhGLU42* and *GhGLU43* was highly specific to roots and leaves. The expression peaks of *GhGLU45* occurred mainly in anthers, which may indicate a role in cotton flowering. At the onset of meiosis, a secondary callose wall composed of β-1,3-glucan is deposited between the primary wall and the plasma membrane of the pollen mother cells^41^. It is reported that the timing of the callose wall degradation by β-1,3-glucanase is important for proper microsporegenesis, and the premature dissolution and delayed degradation of the callose walls caused male sterility in tobacco^9^ and rice^4^, respectively.

The protein domain architecture and intron/exon structure of the genes in subfamily E were highly conserved (Fig. 3). Most of these genes lost the C-terminal region, including the CBM43 domain and the hydrophobic C-terminal sequence, and this change may enable them to be secreted extracellularly. Furthermore, *GLUs* in subfamily E experienced quicker protein evolution in both diploid and polyploid cotton, and showed subgenome-biased expression. Our findings suggest that the specific expression, loss of the C-terminal region, fast evolution and subgenome expression bias of these members together led to the functional divergence of *GLUs* in subfamily E in cotton.

As a member of the pathogenesis-related (PR) protein group, the interest in β-1,3-glucanases has focused primarily on their antifungal activity. This glucanohydrolase can degrade β-1,3-glucan in the cell walls of pathogens, and the release of cell-wall derived elicitors can further stimulate defense reactions^7^,^8^. In soybean, β-1,3-glucanases have been demonstrated to release active elicitors from fungal cell walls, and then induce the accumulation of the phytoalexin glyceollin^42^. Constitutive expression of a β-1,3-glucanase gene in tobacco plants led to reduced symptoms when infected with the glucan-containing fungi *Peronospora tabacina* and *Phytophthora parasiticavar nicotiana*^43^. Further, expression of the β-1,3-glucanase gene in combination with chitinase transgenes led to a remarkable synergic effect. Transgenic tomato plants that were overexpressing β-1,3-glucanase in class I and chitinase from tobacco, showed strong antifungal activity against *Fusarium oxysporum*^44^. Tobacco plants expressing both β-1,3-glucanase and chitinase showed reduced susceptibility to infection by *Fusarium solani*^45^.

*Verticillium* wilt is a serious disease that significantly reduces the yield and quality of cotton. The availability of the whole genome sequences of *Gossypium* species provide us with an opportunity to identify certain *GLUs* that are involved in *V. dahliae* resistance. In this study, based on the genome-wide analyses, we found two tandem duplicated *GLUs* in subfamily E, *GLU42* and *GLU43*, which were significantly induced in cotton roots after *V. dahliae* inoculation. VIGS assays showed that silencing of these two genes significantly increased the susceptibility of cotton plants to *V. dahlia*, while the control and mock treated plants exhibited a weak and partial leaf wilting phenotype (Fig. 10). Our finding implies that *GLU42* and *GLU43* play crucial roles in resistance to *V. dahliae* in cotton, and that overexpression of *GLU42*/*43* will help defend cotton against fungal infection.

## Materials and Methods

### Identification and chromosomal mapping

The gene files of *G. raimondii*, *A. thaliana*, *V. vinifera*, and *T. cacao* were downloaded from Phytozome v9.0 (http://www.phytozome.net/). The gene information of *G. arboreum*, *G. hirsutum* acc. TM-1, and *G. barbadense* acc. 3–79, were downloaded from http://cgp.genomics.org.cn, http://mascotton.njau.edu.cn and http://cotton.cropdb.org, respectively. The Hidden Markov Model (HMM) profile of the glycoside hydrolase family 17 domain (PF00332) was obtained from the Pfam website (http://pfam.xfam.org/), and was employed as a query to identify all possible *GLUs* using HMMER (V3.0) software^46^. To validate the HMM search, all candidate sequences were used as queries to search the NCBI non-redundant (nr) protein database with the blastp program, and the results with the best hits of “Glyco_hydro_17” were retained for further analysis. For prediction of GPI-anchor attachment sites, the BGI-PI^47^ and GPI-SOM^48^ algorithms were used.

Positional information on *GLUs* was parsed from the General Feature Format (GFF) files downloaded from Phytozome v9.0, and the locations of *GLUs* in *G. raimondii* and *G. hirsutum* were drafted by MapInspect software (http://www.plantbreeding.wur.nl/uk/software-mapinspect.html).

### Phylogenetic and exon-intron structural analysis

A multiple alignment of the sequences encoding the conserved glycosyl hydrolase family 17 domain were constructed with ClustalX (version 2.0)^49^, and gaps and poorly aligned sections were removed. A phylogenetic tree was generated using the maximum likelihood method and WAG model in MEGA v5.2^50^ software, and the reliability of interior branches was assessed with 1000 bootstrap resamplings.

The gene structure of the *GLUs* was parsed from the General Feature Format (GFF) files, and diagrams of the exon-intron structures were drawn using the online program Gene Structure Display Server (GSDS; http://gsds.cbi.pku.edu.cn/).

### Genome synteny and gene duplication

The syntenic information of *G. raimondii*, *A. thaliana*, *V. vinifera* and *T. cacao* was downloaded from the Plant Genome Duplication Database (PGDD; http://chibba.agtec.uga.edu/duplication/). *GLUs* were mapped to the syntenic blocks for intra- and inter-genomic comparison. The syntenic diagram was drawn using Circos software^51^.

The timing of segmental duplication events can be estimated by computing mean *Ks* values for all anchor points located in the corresponding syntenic block^16^,^25^, and all the *Ks* values were parsed from PGDD syntenic data. Genes separated by five or fewer genes within a 100-kb region on a chromosome may have resulted from tandem duplication^52^.

### Plant materials and treatments

The genetic standard line, *G. hirsutum* acc. TM-1, was used for tissue/organ expression analysis. Roots, stems and leaves were collected from two-week-old seedlings grown in a greenhouse. Petals, anthers and ovules were collected from plants grown under standard field conditions on the day of flowering, and fibers were excised from developing bolls on selected days post anthesis (DPA). The tissue/organ materials were quick-frozen in liquid nitrogen and stored at −70 °C before RNA extraction.

*G. barbadense* cv. Hai7124 with *Verticillium* resistance, was used for fungal pathogen (*V. dahliae*) inoculation. We hand-inoculated three-week-old Hai7124 seedlings with conidial suspensions carrying 1 × 10^7^ spores of *V. dahliae* strain V991 through dip-infection. The roots were harvested, with three repeats at different time points (0, 24, 48, 96 and 144 hours) after V991 treatment, and then quick-frozen in liquid nitrogen and stored at −70 °C before RNA extraction. *G. hirsutum* cv. Junmian 1 with *Verticillium* susceptibility was used as a susceptible control.

### RNA isolation and quantitative reverse transcription PCR

Total RNA was isolated using the CTAB-acidic Phenolic method^53^. Each RNA sample was treated with DNase I to remove the genomic DNA. Total RNA samples (2 μg per reaction) from different tissues/organ were reversely transcribed into cDNA by M-MLV reverse transcriptase.

The expression of *GLUs* was analyzed using an ABI 7500 real-time PCR system with the HiScript Q RT SuperMix (Vazyme, Nanjing, China). Gene-specific primers were designed based on the β-1,3-glucanase gene sequences using Oligo 6.0. Cotton *histone3* (AF024716) was used as the reference gene^54^. The amplification parameters were as follows: 95 °C hold for 10 min, followed by 40 cycles at 95 °C for 15 s, 58 °C for 15 s and 72 °C for 15 s. For the melting curve stage, the default settings were chosen. Nonspecific products were identified by inspecting melting curves. The primer pairs used for real-time PCR were listed in Supplementary Table 5.

### Estimation of the evolution rates of β-1,3-glucanase genes

Estimation of the rates of nonsynonymous substitutions per nonsynonymous site (*Ka*) and synonymous substitutions per synonymous site (*Ks*) was performed within and between *Gossypium* species using DnaSP version 5^55^. Based on the definition of *Ka/Ks*, a value of 1 represented neutral evolution, and a value less than 1 indicated negative or purifying selection, whereas a value greater than 1 indicated positive selection acting on amino acids.

### Construction of VIGS vectors and agro-infiltration

The binary TRV vectors pTRV1 and pTRV2 were kindly provided by Dr. Libo Shan at Texas A&M University (College Station, TX, USA). pTRV2:*GhCLA1*, where *GhCLA1* (Cloroplastos alterados 1) encodes 1-deoxy-D-xylulose-5-phosphate synthase, was used as a control^56^. The sequences of *GLU42* and *GLU43* were amplified using the primers listed in Supplementary Table 5, and the PCR fragments cloned from Hai7124, with 334 bp for *GLU42* and 326 bp for *GLU43*, were inserted into pTRV2. These pYL156 derivatives were individually transformed into Agrobacterium GV3101.

Agrobacterium cultures containing recombinant TRV vectors were prepared and then infiltrated into two fully expanded cotyledons of eight-day-old seedlings, as previously described by Zhang *et al*.^57^. The seedlings were cultivated in a greenhouse at 23/22 °C (day/night) with a 16 h light/8 h dark photoperiod. Agrobacterium-mediated VIGS assays were repeated three times with more than 20 plants for each construct per repeat.

## Additional Information

**How to cite this article**: Xu, X. *et al*. Genome-wide characterization of the β-1,3-glucanase gene family in *Gossypium* by comparative analysis. *Sci. Rep.* **6**, 29044; doi: 10.1038/srep29044 (2016).

## Supplementary Material

Supplementary Information

## Figures and Tables

**Table 1:** Size of the five protein domain architectures of β-1,3-glucanases in different species.

Species name	Total	I	II	III	IV	V
*Gossypium raimondii*	67	23	15	1	19	9
*Arabidopsis thaliana*	51	19	9	1	10	12
*Theobroma cacao*	44	16	10	0	7	10
*Vitis vinifera*	43	9	10	0	12	8

The number of β-1,3-glucanases in each protein domain architecture is listed in *G. raimondii*, *A. thaliana*, *T. cacao* and *V. vinifera*. Thecc1EG027171t1 in *T. cacao* and GSVIVT01030073001, GSVIVT01012711001, GSVIVT01022253001, and GSVIVT01025431001 in *V. vinifera* had not classified into the above five types due to the existence of other domains in C-terminal.

**Table 2:** Ks values of gene pairs in syntenic blocks.

Gene 1	Gene 2	Anchors*	Ks		Gene 1	Gene 2	Anchors*	Ks	
			Mean	SD				Mean	SD
*GrGLU56*	*GrGLU61*	65	0.806	0.343	*GrGLU11*	*GrGLU41*	54	0.771	0.383
*GrGLU56*	*GrGLU39*	15	0.713	0.294	*GrGLU3*	*GrGLU5*	34	0.557	0.206
*GrGLU39*	*GrGLU61*	56	0.634	0.338	*GrGLU57*	*GrGLU63*	32	0.436	0.119
*GrGLU14*	*GrGLU31*	34	0.684	0.460	*GrGLU67*	*GrGLU34*	15	0.631	0.367
*GrGLU15*	*GrGLU49*	55	0.605	0.418	*GrGLU4*	*GrGLU48*	35	0.701	0.360
*GrGLU24*	*GrGLU30*	9	0.593	0.213	*GrGLU62*	*GrGLU55*	22	0.706	0.335
*GrGLU1*	*GrGLU19*	33	0.701	0.411					
*GrGLU26*	*GrGLU41*	27	0.718	0.257	*GrGLU30*	*GrGLU50*	9	2.034	0.232
*GrGLU26*	*GrGLU11*	115	0.534	0.289	*GrGLU6*	*GrGLU17*	15	2.260	1.016

^*^Numbers of gene pairs in syntenic blocks.

**Table 3:** Molecular evolutionary rates of β-1,3-glucanase genes in various comparisons among *Gossypium* taxa.

Subfamily	Ka/Ks/Ka:Ks ratio			
	A vs At^a^	D vs Dt	A vs D	At vs Dt
A	0.0026/0.0077/0.3469	0.0037/0.0120/0.5216	0.0088/0.0412/0.2368	0.0079/0.0398/0.2371
B	0.0033/0.0109/0.3281	0.0042/0.0226/0.2055	0.0098/0.0590/0.1582	0.0115/0.0455/0.2577
C	0.0031/0.0098/0.5355	0.0027/0.0124/0.3123	0.0106/0.0496/0.2201	0.0097/0.0506/0.1900
D	0.0035/0.0037/0.7288	0.0041/0.0105/0.3837	0.0110/0.0487/0.2152	0.0112/0.0467/0.2259
E	0.0044*/0.0107/0.4989	0.0063*/0.0156/0.4986	0.0162*/0.0489/0.3312*	0.016*2/0.0503/0.3305*
F	0.0019/0.0081/0.2733	0.0033/0.0148/0.3064	0.0095/0.0455/0.2301	0.0103/0.0436/0.2406
G	0.0032/0.0112/0.4037	0.0032/0.0134/0.2446	0.0090/0.0506/0.1788	0.0090/0.0505/0.1746
H	0.0028/0.0136/0.2855	0.0036/0.0133/0.6975	0.0049/0.0417/0.1607	0.0071/0.0355/0.2927

^a^Genome codes are as follows: *G. arboreum* (A), *G. raimondii* (D), *G. hirsutum* “A homoelog” (At), *G. hirsutum* “D homoeolog” (Dt).

“*”: t test at P < 0.05.
