# Supplementary material for: Genome-wide characterization of the β-1,3-glucanase gene family in Gossypium by comparative analysis
Source: Sci Rep. 2016 Jun 29;6:29044. doi: 10.1038/srep29044 (PMC4926093; doi:10.1038/srep29044)
Supplement: Supplementary Information [file srep29044-s1.pdf]

Genome-wide characterization of the β-1,3-glucanase gene family in *Gossypium* by comparative analysis

Xiaoyang Xu[1], Yue Feng[1], Shuai Fang[1], Jun Xu[1], Xinyu Wang[2*], Wangzhen Guo[1]*

[1]State Key Laboratory of Crop Genetics & Germplasm Enhancement, Hybrid Cotton R & D Engineering Research Center, MOE, Nanjing Agricultural University, Nanjing 210095, China

[2]College of Life Science, Nanjing Agricultural University, Nanjing 210095, China

*** Correspondence and requests for materials should be addressed to W.Z.G or X.Y.W. (email: moelab@njau.edu.cn; xywang@njau.edu.cn)**

Supplementary Figures and Tables

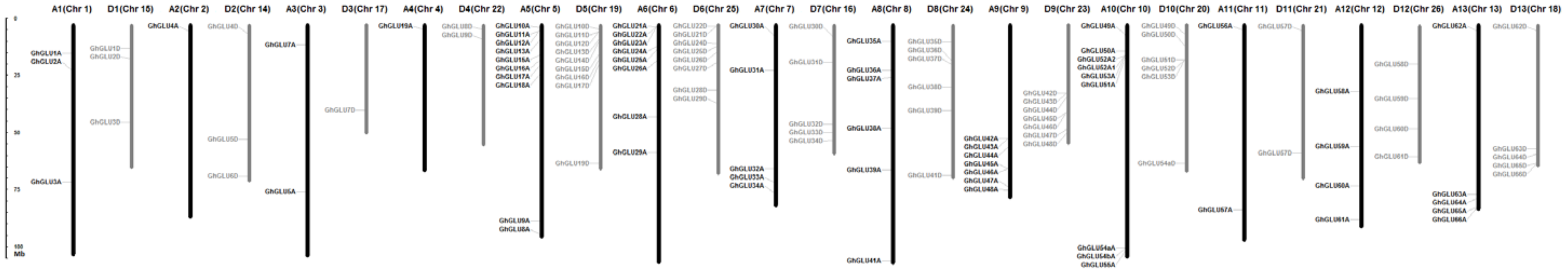

Supplementary Figure 1. Chromosomal distribution of β-1,3-glucanase genes in *G. hirsutum*. Genes in A subgenome chromosomes were in black, and in D subgenome chromosomes in gray.

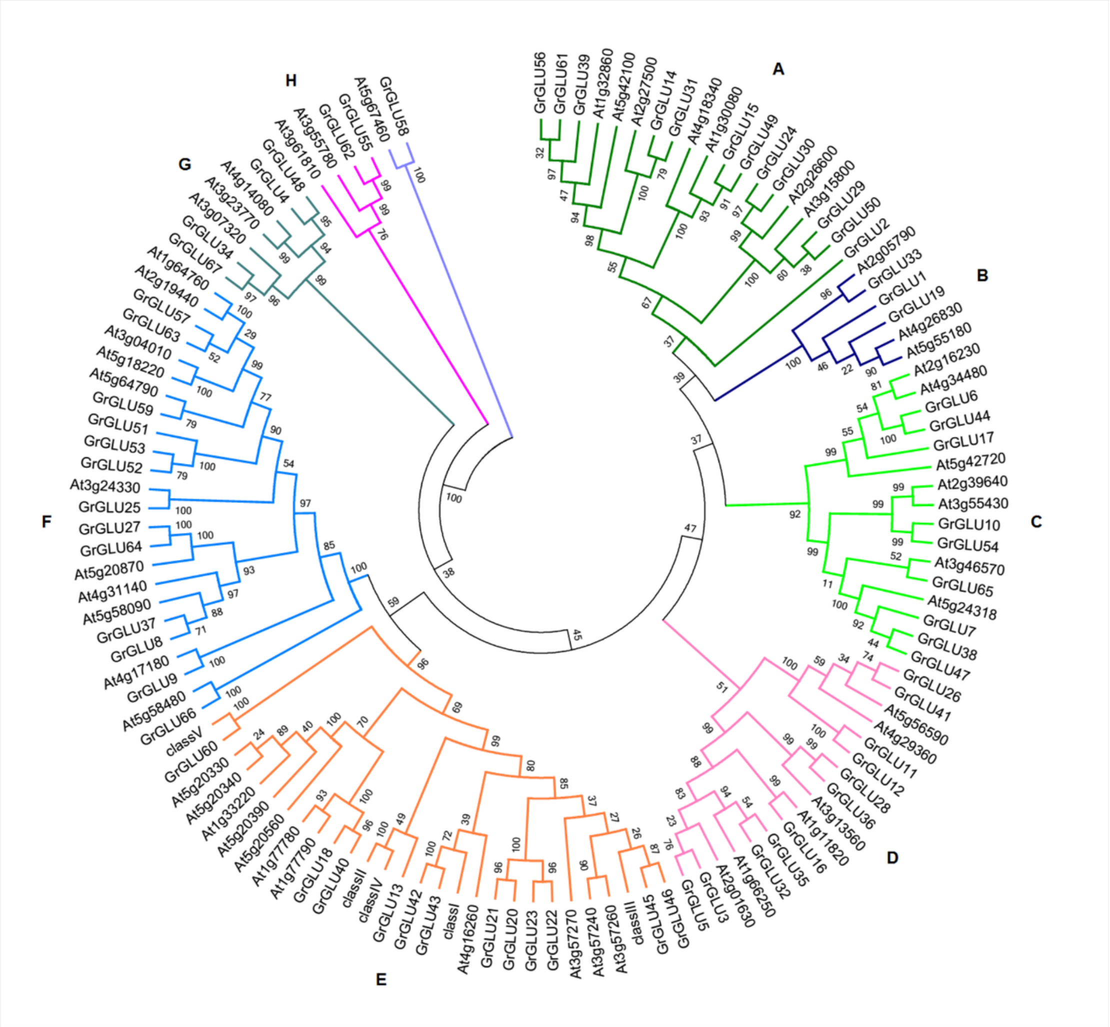

Supplementary Figure 2. Phylogenetic relationships of β-1,3-glucanase gene family in *G. raimondii*, *A. thaliana* and tobacco. Amino acid sequences of conserved glycosyl hydrolase family 17 domain were aligned using ClustalX and gaps and poorly aligned sections were removed. Phylogenetic tree was generated using the ML method under WAG model in MEGA v5.2, and the reliability of interior branches was assessed with 1000 bootstrap resamplings.

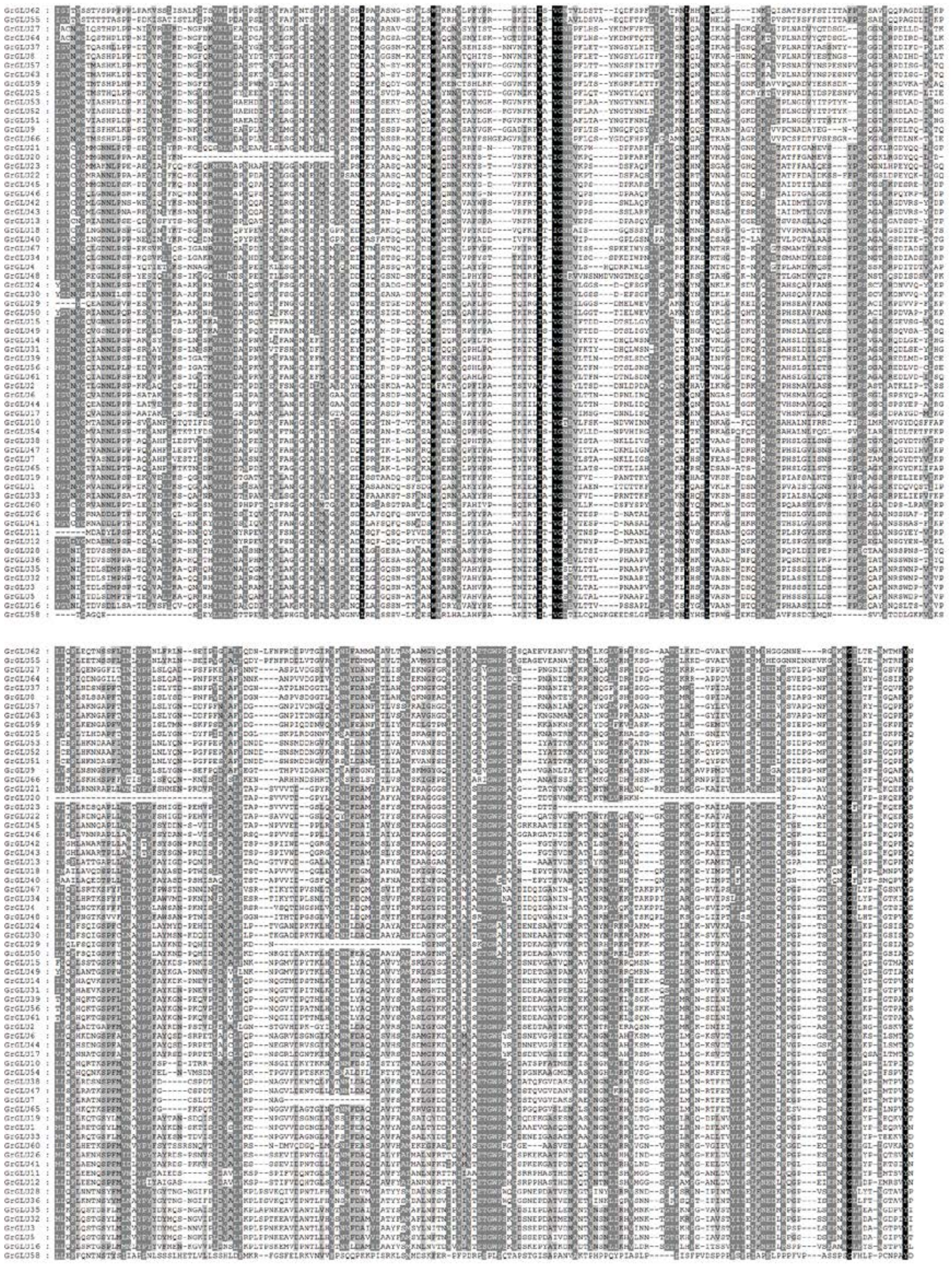

Supplementary Figure 3. The multiple alignment of the conserved glycosyl hydrolase family 17 domain.

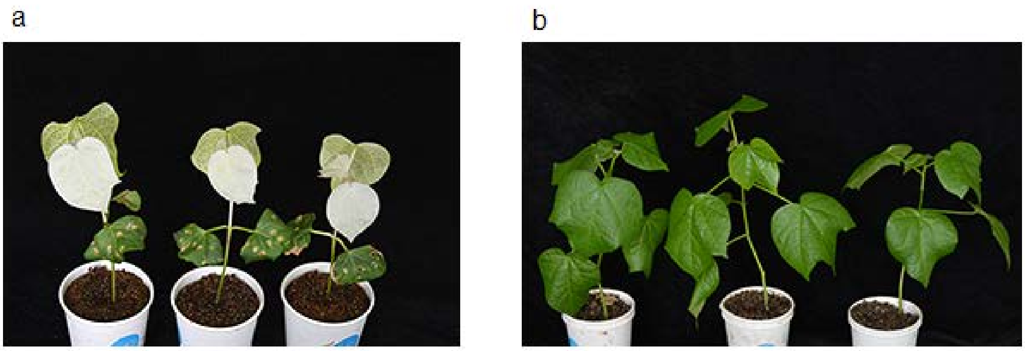

Supplementary Figure 4. Phenotype of target gene-silenced plants.

Photobleaching phenotype of *CLA1* silenced plants (a) and normal phenotype of no-inoculation control of *GLU42/43* silenced plants (b).

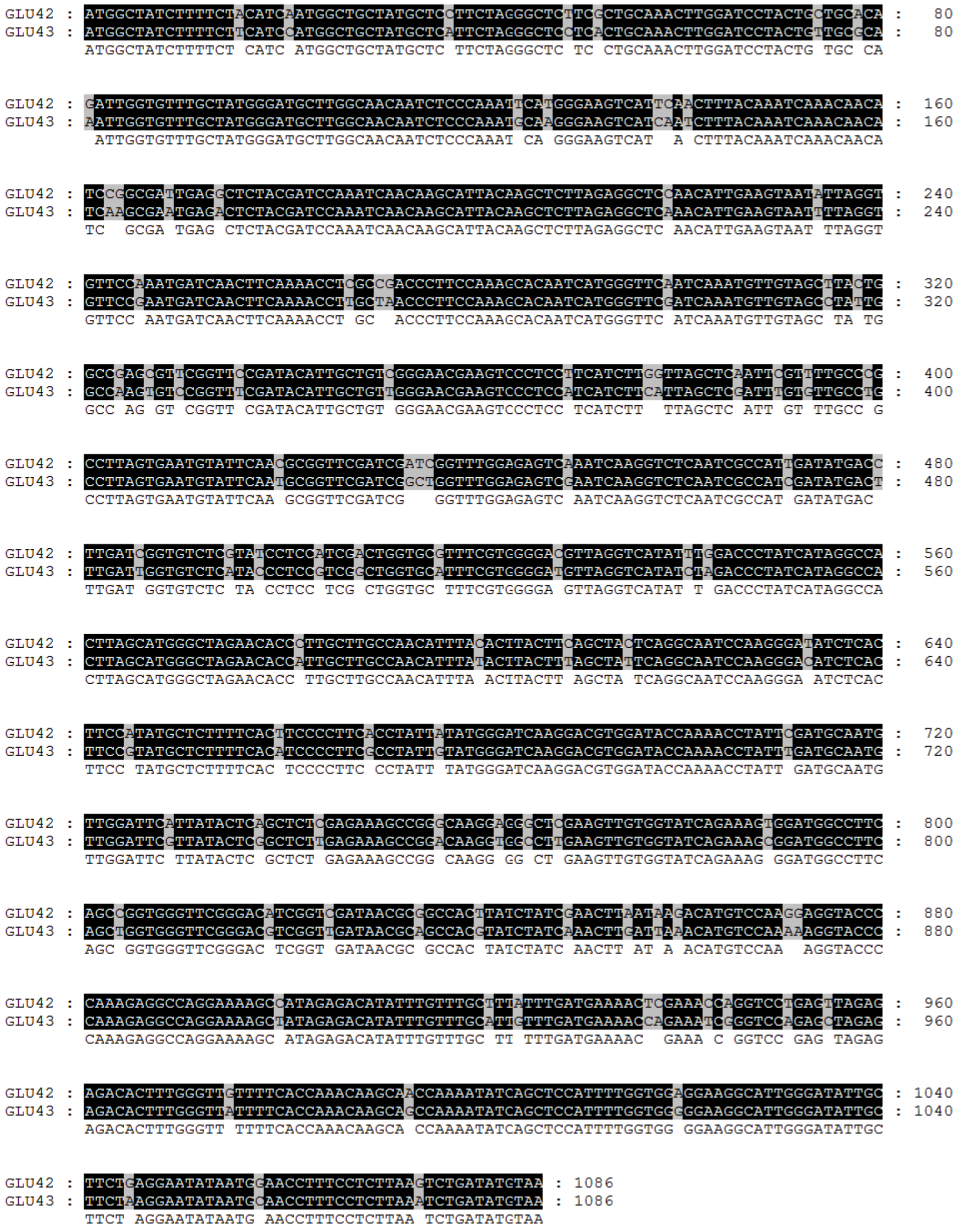

Supplementary Figure 5. Comparison of the nucleotide sequences of *GrGLU42* and *GrGLU43*.

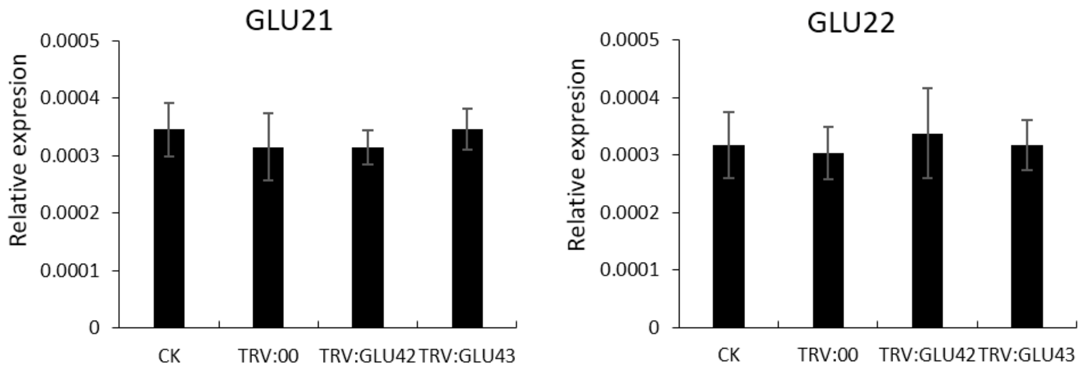

Supplementary Figure 6. Expression of *GLU21/22* in control groups and *GLU42/43* silenced plants.

Supplementary Table 1. β-1,3-glucanase genes in *Gossypium*.

G.arboreum	Locus name	*G. hirsutum* A subgenome	Locus name	*G. barbadense* A subgenome	Locus name	*G.raimondii*	Locus name	*G. hirsutum* D subgenome	Locus name	*G. barbadense* D subgenome	Locus name
GaGLU1	Cotton_A_04825	GhGLU1A	Gh_A01G0649	GbGLU1A1	Gbscaffold1521.13.0	GrGLU1	Gorai.002G092600	GhGLU1D	Gh_D01G0668	GbGLU1D	Gbscaffold6144.12.0
				GbGLU1A2	Gbscaffold1521.12.0						
GaGLU2	Cotton_A_12674	GhGLU2A	Gh_A01G0828			GrGLU2	Gorai.002G114200	GhGLU2D	Gh_D01G0856	GbGLU2D	Gbscaffold12440.10.0
GaGLU3	Cotton_A_36373	GhGLU3A	Gh_A01G1227	GbGLU3A	Gbscaffold620.3.0	GrGLU3	Gorai.002G174100	GhGLU3D	Gh_D01G1411	GbGLU3D	Gbscaffold24022.4.0
GaGLU4	Cotton_A_25304	GhGLU4A	Gh_A02G0175	GbGLU4A	Gbscaffold1889.1.0	GrGLU4	Gorai.005G026800	GhGLU4D	Gh_D02G0239	GbGLU4D	Gbscaffold41178.2.0
GaGLU5	Cotton_A_29885	GhGLU5A	Gh_A03G1035	GbGLU5A	Gbscaffold14449.2.0	GrGLU5	Gorai.005G159100	GhGLU5D	Gh_D02G1427	GbGLU5D	Gbscaffold9656.3.0
GaGLU6	Cotton_A_00745	GhGLU6A	Gh_A03G2183	GbGLU6A1	Gbscaffold5424.6.0	GrGLU6	Gorai.005G243100	GhGLU6D	Gh_D02G2139	GbGLU6D	Gbscaffold20579.3.0
				GbGLU6A2	Gbscaffold5424.5.0						
GaGLU7	Cotton_A_20326	GhGLU7A	Gh_A03G0416	GbGLU7A	Gbscaffold69.15.0	GrGLU7	Gorai.003G121900	GhGLU7D	Gh_D03G1098	GbGLU7D	Gbscaffold7000.1.0
GaGLU8	Cotton_A_06068	GhGLU8A	Gh_A05G3538	GbGLU8A	Gbscaffold2336.19.0	GrGLU8	Gorai.012G009200	GhGLU8D	Gh_D04G0069	GbGLU8D	Gbscaffold22319.4.0
GaGLU9	Cotton_A_17576	GhGLU9A	Gh_A05G3256	GbGLU9A	Gbscaffold2048.19.0	GrGLU9	Gorai.012G043900	GhGLU9D	Gh_D04G0353	GbGLU9D	Gbscaffold13974.24.0
GaGLU10	Cotton_A_11521	GhGLU10A	Gh_A05G0033	GbGLU10A1	Gbscaffold5097.11.0	GrGLU10	Gorai.009G010800	GhGLU10D	Gh_D05G0088	GbGLU10D	Gbscaffold8119.3.0
				GbGLU10A2	Gbscaffold5097.12.0						
GaGLU11	Cotton_A_09400	GhGLU11A	Gh_A05G0189	GbGLU11A	Gbscaffold26219.2.0	GrGLU11	Gorai.009G028200	GhGLU11D	Gh_D05G0263	GbGLU11D1	Gbscaffold6004.3.0
										GbGLU11D2	Gbscaffold6004.4.0
GaGLU12	Cotton_A_09399	GhGLU12A	Gh_A05G0190	GbGLU12A1	Gbscaffold26219.4.0	GrGLU12	Gorai.009G028300	GhGLU12D	Gh_D05G0264	GbGLU12D1	Gbscaffold6004.6.0
				GbGLU12A2	Gbscaffold26219.3.0					GbGLU12D2	Gbscaffold6004.5.0
GaGLU13	Cotton_A_01055	GhGLU13A	Gh_A05G0579	GbGLU13A	Gbscaffold648.10.0	GrGLU13	Gorai.009G074100	GhGLU13D	Gh_D05G0709	GbGLU13D	Gbscaffold13680.4.0
GaGLU14	Cotton_A_13878			GbGLU14A	Gbscaffold17791.1.0	GrGLU14	Gorai.009G085300	GhGLU14D	Gh_D05G0815	GbGLU14D1	Gbscaffold4759.40.0
										GbGLU14D2	Gbscaffold4759.39.0
GaGLU15	Cotton_A_12341	GhGLU15A	Gh_A05G1246	GbGLU15A	Gbscaffold2710.8.0	GrGLU15	Gorai.009G154800	GhGLU15D	Gh_D05G1409	GbGLU15D1	Gbscaffold33334.8.0
										GbGLU15D2	Gbscaffold33334.7.0
GaGLU16	Cotton_A_04676	GhGLU16A	Gh_A05G1462	GbGLU16A	Gbscaffold497.45.0	GrGLU16	Gorai.009G179400	GhGLU16D	Gh_D05G1636	GbGLU16D	Gbscaffold10982.20.0
GaGLU17	Cotton_A_13748	GhGLU17A	Gh_A05G1670	GbGLU17A	Gbscaffold15959.26.0	GrGLU17	Gorai.009G202900	GhGLU17D	Gh_D05G1854	GbGLU17D	Gbscaffold803.34.0
GaGLU18	Cotton_A_16131	GhGLU18A	Gh_A05G1996	GbGLU18A	Gbscaffold999.18.0	GrGLU18	Gorai.009G242500	GhGLU18D	Gh_D05G3800	GbGLU18D	Gbscaffold43820.2.0
GaGLU19	Cotton_A_12137	GhGLU19A	Gh_A04G0109	GbGLU19A	Gbscaffold1811.1.0	GrGLU19	Gorai.009G442700	GhGLU19D	Gh_D05G3612	GbGLU19D	Gbscaffold13331.11.0
GaGLU20	Cotton_A_18246					GrGLU20	Gorai.010G003400			GbGLU20D1	Gbscaffold17324.19.0
										GbGLU20D2	Gbscaffold17324.18.0
GaGLU21	Cotton_A_18247	GhGLU21A	Gh_A06G0035	GbGLU21A	Gbscaffold7493.26.0	GrGLU21	Gorai.010G003500	GhGLU21D	Gh_D06G2278	GbGLU21D	Gbscaffold17324.17.0
GaGLU22	Cotton_A_18248	GhGLU22A	Gh_A06G0036	GbGLU22A	Gbscaffold7493.27.0	GrGLU22	Gorai.010G003600	GhGLU22D	Gh_D06G2277	GbGLU22D	Gbscaffold17324.16.0

GaGLU23	Cotton_A_18249	GhGLU23A	Gh_A06G0037	GbGLU23A	Gbscaffold7493.28.0	GrGLU23	Gorai.010G003700			GbGLU23D	Gbscaffold17324.15.0
GaGLU24	Cotton_A_17417	GhGLU24A	Gh_A06G0470	GbGLU24A	Gbscaffold4129.4.0	GrGLU24	Gorai.010G062700	GhGLU24D	Gh_D06G0512		
GaGLU25	Cotton_A_20448	GhGLU25A	Gh_A06G0517	GbGLU25A	Gbscaffold3655.6.0	GrGLU25	Gorai.010G068800	GhGLU25D	Gh_D06G0574	GbGLU25D1	Gbscaffold11193.8.0
										GbGLU25D2	Gbscaffold11193.9.0
GaGLU26	Cotton_A_18097	GhGLU26A	Gh_A06G0594	GbGLU26A	Gbscaffold2850.13.0	GrGLU26	Gorai.010G078300	GhGLU26D	Gh_D06G0675	GbGLU26D	Gbscaffold18465.3.0
GaGLU27	Cotton_A_36325	GhGLU27A	Gh_A06G1946	GbGLU27A1	Gbscaffold9289.2.0	GrGLU27	Gorai.010G094500	GhGLU27D	Gh_D06G0862	GbGLU27D1	Gbscaffold6999.20.0
				GbGLU27A2	Gbscaffold9289.1.0					GbGLU27D2	Gbscaffold6999.23.0
GaGLU28	Cotton_A_26811	GhGLU28A	Gh_A06G0935			GrGLU28	Gorai.010G126400	GhGLU28D	Gh_D06G1164	GbGLU28D	Gbscaffold10174.1.0
GaGLU29	Cotton_A_38959	GhGLU29A	Gh_A06G1032			GrGLU29	Gorai.010G137300	GhGLU29D	Gh_D06G1248		
GaGLU30	Cotton_A_03523	GhGLU30A	Gh_A07G0310	GbGLU30A1	Gbscaffold7622.14.0	GrGLU30	Gorai.001G043100	GhGLU30D	Gh_D07G0367		Gbscaffold80.10.0
				GbGLU30A2	Gbscaffold7622.13.0						
GaGLU31	Cotton_A_09592	GhGLU31A	Gh_A07G1004	GbGLU31A	Gbscaffold21225.4.0	GrGLU31	Gorai.001G122300	GhGLU31D	Gh_D07G1081	GbGLU31D1	Gbscaffold2759.2.0
										GbGLU31D2	Gbscaffold2759.3.0
GaGLU32	Cotton_A_39010	GhGLU32A	Gh_A07G1597	GbGLU32A	Gbscaffold33713.1.0	GrGLU32	Gorai.001G204900	GhGLU32D	Gh_D07G1793	GbGLU32D	Gbscaffold12719.9.0
GaGLU33	Cotton_A_18690	GhGLU33A	Gh_A07G1684	GbGLU33A1	Gbscaffold31935.1.0	GrGLU33	Gorai.001G216900	GhGLU33D	Gh_D07G1895	GbGLU33D	Gbscaffold8463.10.0
				GbGLU33A2	Gbscaffold31935.2.0						
GaGLU34	Cotton_A_15198	GhGLU34A	Gh_A07G1802	GbGLU34A	Gbscaffold7792.2.0	GrGLU34	Gorai.001G230200	GhGLU34D	Gh_D07G2007	GbGLU34D	Gbscaffold10953.5.0
GaGLU35	Cotton_A_03296	GhGLU35A	Gh_A08G0480	GbGLU35A	Gbscaffold6441.5.0	GrGLU35	Gorai.004G064200	GhGLU35D	Gh_D08G0563	GbGLU35D	Gbscaffold400.15.0
GaGLU36	Cotton_A_24254	GhGLU36A	Gh_A08G0722	GbGLU36A	Gbscaffold2932.3.0	GrGLU36	Gorai.004G094500	GhGLU36D	Gh_D08G0842	GbGLU36D	Gbscaffold7397.3.0
GaGLU37	Cotton_A_35998	GhGLU37A	Gh_A08G0738	GbGLU37A	Gbscaffold3293.1.0	GrGLU37	Gorai.004G097500	GhGLU37D	Gh_D08G0877	GbGLU37D	Gbscaffold3546.10.0
GaGLU38	Cotton_A_36462	GhGLU38A	Gh_A08G0841	GbGLU38A	Gbscaffold4788.2.0	GrGLU38	Gorai.004G111800	GhGLU38D	Gh_D08G1019	GbGLU38D1	Gbscaffold3463.6.0
										GbGLU38D2	Gbscaffold3463.7.0
GaGLU39	Cotton_A_34954	GhGLU39A	Gh_A08G0958	GbGLU39A1	Gbscaffold866.5.0	GrGLU39	Gorai.004G128800	GhGLU39D	Gh_D08G1168	GbGLU39D1	Gbscaffold2085.8.0
				GbGLU39A2	Gbscaffold866.9.0					GbGLU39D2	Gbscaffold2085.10.0
				GbGLU39A3	Gbscaffold866.7.0						
GaGLU40	Cotton_A_05737			GbGLU40A1	Gbscaffold17750.12.0	GrGLU40	Gorai.004G257700	GhGLU40D	Gh_Sca008361G02	GbGLU40D	Gbscaffold7703.10.0
				GbGLU40A2	Gbscaffold17750.11.0						
GaGLU41	Cotton_A_14534	GhGLU41A	Gh_A08G2169	GbGLU41A	Gbscaffold9174.22.0	GrGLU41	Gorai.004G282300	GhGLU41D	Gh_D08G2535	GbGLU41D	Gbscaffold18520.31.0
GaGLU42	Cotton_A_16539	GhGLU42A	Gh_A09G0635	GbGLU42A	Gbscaffold7561.11.0	GrGLU42	Gorai.006G079400	GhGLU42D	Gh_D09G0636	GbGLU42D1	Gbscaffold17082.1.0
										GbGLU42D2	Gbscaffold17082.2.0
GaGLU43	Cotton_A_16537	GhGLU43A	Gh_A09G0636	GbGLU43A1	Gbscaffold7561.13.0	GrGLU43	Gorai.006G079600	GhGLU43D	Gh_D09G0637		
				GbGLU43A2	Gbscaffold7561.14.0						
				GbGLU43A3	Gbscaffold7561.15.0						
GaGLU44	Cotton_A_35086	GhGLU44A	Gh_A09G0725	GbGLU44A	Gbscaffold1825.2.0	GrGLU44	Gorai.006G090600	GhGLU44D	Gh_D09G0724	GbGLU44D	Gbscaffold1825.2.0

GaGLU45	Cotton_A_01892	GhGLU45A	Gh_A09G1126			GrGLU45	Gorai.006G134600	GhGLU45D	Gh_D09G1130	GbGLU45D1	Gbscaffold33965.9.0
										GbGLU45D2	Gbscaffold33965.8.0
GaGLU46	Cotton_A_01893	GhGLU46A	Gh_A09G1127	GbGLU46A	Gbscaffold33965.10.0	GrGLU46	Gorai.006G134700	GhGLU46D	Gh_D09G1131	GbGLU46D	Gbscaffold8357.31.0
GaGLU47	Cotton_A_08100	GhGLU47A	Gh_A09G1662	GbGLU47A	Gbscaffold13148.1.0	GrGLU47	Gorai.006G202300	GhGLU47D	Gh_D09G1756	GbGLU47D	Gbscaffold2786.5.0
GaGLU48	Cotton_A_07399	GhGLU48A	Gh_A09G1852	GbGLU48A	Gbscaffold3157.7.0	GrGLU48	Gorai.006G224700	GhGLU48D	Gh_D09G1972	GbGLU48D1	Gbscaffold5795.35.0
										GbGLU48D2	Gbscaffold5795.37.0
GaGLU49	Cotton_A_02897	GhGLU49A	Gh_A10G0385	GbGLU49A	Gbscaffold7446.7.0	GrGLU49	Gorai.011G044700	GhGLU49D	Gh_D10G0396	GbGLU49D	Gbscaffold24196.2.0
GaGLU50	Cotton_A_11774	GhGLU50A	Gh_A10G0654	GbGLU50A1	Gbscaffold5654.2.0	GrGLU50	Gorai.011G084000	GhGLU50D	Gh_D10G0738	GbGLU50D	Gbscaffold7228.4.0
				GbGLU50A2	Gbscaffold11052.10.0						
GaGLU51	Cotton_A_31039	GhGLU51A	Gh_A10G0751	GbGLU51A	Gbscaffold7121.1.0	GrGLU51	Gorai.011G114400	GhGLU51D	Gh_D10G1013	GbGLU51D	Gbscaffold6226.6.0
GaGLU52	Cotton_A_25072	GhGLU52A1	Gh_A10G0737			GrGLU52	Gorai.011G114500	GhGLU52D	Gh_D10G1014	GbGLU52D1	Gbscaffold16259.1.0
		GhGLU52A2	Gh_A10G0736	GbGLU52A2	Gbscaffold51250.1.0					GbGLU52D2	Gbscaffold16259.2.0
										GbGLU52D3	Gbscaffold16259.5.0
GaGLU53	Cotton_A_25071	GhGLU53A	Gh_A10G0738		Gbscaffold4636.5.0	GrGLU53	Gorai.011G114600	GhGLU53D	Gh_D10G1015		
GaGLU54a	Cotton_A_23374	GhGLU54aA	Gh_A10G1964	GbGLU54aA1	Gbscaffold16242.9.0	GrGLU54	Gorai.011G261000	GhGLU54aD	Gh_D10G2246	GbGLU54aD	Gbscaffold19666.7.0
				GbGLU54aA2	Gbscaffold16242.10.0						
GaGLU54b	Cotton_A_23372	GhGLU54bA	Gh_A10G1965	GbGLU54bA	Gbscaffold16242.11.0						
GaGLU55	Cotton_A_13550	GhGLU55A	Gh_A10G2058	GbGLU55A	Gbscaffold20109.3.0	GrGLU55	Gorai.011G279200	GhGLU55D	Gh_D10G2470	GbGLU55D	Gbscaffold21303.10.0
GaGLU56	Cotton_A_02355	GhGLU56A	Gh_A11G0157	GbGLU56A	Gbscaffold9746.17.0	GrGLU56	Gorai.007G018900	GhGLU56D	Gh_D11G0171	GbGLU56D	Gbscaffold7684.25.0
GaGLU57	Cotton_A_15022	GhGLU57A	Gh_A11G2336	GbGLU57A	Gbscaffold16846.12.0	GrGLU57	Gorai.007G287700	GhGLU57D	Gh_D11G2650	GbGLU57D	Gbscaffold744.11.0
GaGLU58	Cotton_A_15376	GhGLU58A	Gh_A12G0700	GbGLU58A	Gbscaffold10231.1.0	GrGLU58	Gorai.008G082600	GhGLU58D	Gh_D12G0713	GbGLU58D	Gbscaffold317.1.0
GaGLU59	Cotton_A_37357	GhGLU59A	Gh_A12G0830	GbGLU59A1	Gbscaffold2804.3.0	GrGLU59	Gorai.008G101400	GhGLU59D	Gh_D12G0895	GbGLU59D1	Gbscaffold2008.13.0
				GbGLU59A2	Gbscaffold2804.2.0					GbGLU59D2	Gbscaffold2008.12.0
GaGLU60	Cotton_A_23048	GhGLU60A	Gh_A12G1321	GbGLU60A	Gbscaffold8011.12.0	GrGLU60	Gorai.008G159300	GhGLU60D	Gh_D12G1446	GbGLU60D	Gbscaffold2654.4.0
GaGLU61	Cotton_A_13195	GhGLU61A	Gh_A12G2182			GrGLU61	Gorai.008G260000	GhGLU61D	Gh_D12G2362		
GaGLU62	Cotton_A_00968	GhGLU62A	Gh_A13G0160	GbGLU62A	Gbscaffold18390.7.0	GrGLU62	Gorai.013G019700	GhGLU62D	Gh_D13G0179	GbGLU62D1	Gbscaffold4634.26.0
										GbGLU62D2	Gbscaffold4634.28.0
GaGLU63	Cotton_A_22413	GhGLU63A	Gh_A13G1509	GbGLU63A	Gbscaffold4625.17.0	GrGLU63	Gorai.013G202800	GhGLU63D	Gh_D13G1839	GbGLU63D1	Gbscaffold11306.7.0
										GbGLU63D2	Gbscaffold11306.8.0
GaGLU64	Cotton_A_03187	GhGLU64A	Gh_A13G1667	GbGLU64A	Gbscaffold6297.3.0	GrGLU64	Gorai.013G221700	GhGLU64D	Gh_D13G2031	GbGLU64D	Gbscaffold15128.8.0
GaGLU65	Cotton_A_10553	GhGLU65A	Gh_A13G1979	GbGLU65A	Gbscaffold21181.8.0	GrGLU65	Gorai.013G263800	GhGLU65D	Gh_D13G2378	GbGLU65D	Gbscaffold17429.3.0
GaGLU66	Cotton_A_10484	GhGLU66A	Gh_A13G2048	GbGLU66A	Gbscaffold26646.15.0	GrGLU66	Gorai.013G270800	GhGLU66D	Gh_D13G2448	GbGLU66D1	Gbscaffold23517.6.0
										GbGLU66D2	Gbscaffold23517.5.0
GaGLU67	Cotton_A_39233	GhGLU67A	Gh_A07G2231	GbGLU67A	Gbscaffold44.5.0	GrGLU67	Gorai.N004800			GbGLU67D	Gbscaffold3704.2.0

Supplementary Table 2. Intra- or inter-species synteny of β-1,3-glucanase genes in *G. raimondii* and other species.

Subfamily	Gene name	Locus name	0.3<Ks<1.5*	Ks>1.5*	At	Tc	Vv
**A**	GrGLU56	Gorai.007G018900	Gorai.004G128800,Gorai.008G260000,GR.06		AT1G32860(1.764)	Thecc1EG015614(0.621)	GSVIVT01019678001(1.593)
	GrGLU61	Gorai.008G260000	Gorai.004G128800,Gorai.007G018900,GR.06	GR.04,GR.08	AT1G32860(2.223)	Thecc1EG015614(0.555)	GSVIVT01019678001(1.436)
	GrGLU39	Gorai.004G128800	Gorai.007G018900,Gorai.008G260000,GR.06	GR.08	AT1G32860(2.002)	Thecc1EG015614(0.422)	GSVIVT01019678001(1.329)
	GrGLU14	Gorai.009G085300	Gorai.001G122300,GR.04,GR.06,GR.09	GR.02	AT2G27500(1.852)	Thecc1EG041403(0.468)	GSVIVT01036124001(1.233)
	GrGLU31	Gorai.001G122300	Gorai.009G085300,GR.02,GR.04,GR.06		AT2G27500(1.695)	Thecc1EG041403(0.457)	GSVIVT01036124001(1.417)
	GrGLU15	Gorai.009G154800	Gorai.011G044700,GR.06,GR.10,GR.10		AT1G30080(1.890)	Thecc1EG029478(0.433)	GSVIVT01021499001(1.333)
					AT4G18340(1.791)		
	GrGLU49	Gorai.011G044700	Gorai.009G154800,GR.10,GR.10,GR.06		AT1G30080(1.955)	Thecc1EG029478(0.422)	GSVIVT01021499001(1.381)
					AT4G18340(1.624)		
	GrGLU24	Gorai.010G062700	Gorai.001G043100,GR.13,GR.09	GR.09		Thecc1EG038109(0.444)	GSVIVT01015563001(1.200)
	GrGLU30	Gorai.001G043100	Gorai.010G062700,GR.04,GR.09	Gorai.011G084000		Thecc1EG038109(0.415)	GSVIVT01015563001(1.025)
	GrGLU29	Gorai.010G137300					
	GrGLU50	Gorai.011G084000	GR.10	Gorai.001G043100,GR.10	AT3G15800(1.859)	Thecc1EG027171(0.556)	GSVIVT01017331001(1.227)
						Thecc1EG038109(1.747)	
	GrGLU2	Gorai.002G114200	GR.05,GR.09			Thecc1EG034422(0.431)	GSVIVT01013401001(1.232)
**B**	GrGLU33	Gorai.001G216900	GR.01,GR.04,GR.07	GR.09		Thecc1EG002168(0.461)	GSVIVT01030503001(1.362)
							GSVIVT01037711001(2.517)
	GrGLU1	Gorai.002G092600	Gorai.009G442700,GR.13	GR.11,GR.07		Thecc1EG030805(0.474)	GSVIVT01037711001(1.325)
	GrGLU19	Gorai.009G442700	Gorai.002G092600,GR.13			Thecc1EG030805(0.607)	GSVIVT01037711001(1.290)
**C**	GrGLU6	Gorai.005G243100	GR.13,GR.08	Gorai.009G202900		Thecc1EG006900(0.617)	GSVIVT01009700001(2.146)
						Thecc1EG034024(2.085)	GSVIVT01031959001(1.259)
	GrGLU44	Gorai.006G090600	GR.11,				
	GrGLU17	Gorai.009G202900	GR.02,GR.05,GR.10,GR.09	Gorai.005G243100,GR.03,GR.08		Thecc1EG034024(0.448)	GSVIVT01009700001(1.275)
						Thecc1EG006900(1.364)	
	GrGLU10	Gorai.009G010800	GR.04			Thecc1EG038357(0.457)	
	GrGLU54	Gorai.011G261000	GR.12	GR.13,GR.03,GR.06	AT3G55430(1.995)		GSVIVT01016553001(1.403)
							GSVIVT01024956001(1.869)

	GrGLU65	Gorai.013G263800	GR.01,GR.13,GR.09	GR.11,GR.12,GR.03		Thecc1EG041833(0.703)	GSVIVT01024956001(1.396)
						Thecc1EG042768(1.692)	
	GrGLU7	Gorai.003G121900	GR.08			Thecc1EG015448(0.526)	GSVIVT01038583001(1.747)
	GrGLU38	Gorai.004G111800					
	GrGLU47	Gorai.006G202300	GR.07,GR.08			Thecc1EG015448(0.496)	GSVIVT01038583001(1.096)
**D**	GrGLU26	Gorai.010G078300	Gorai.004G282300,Gorai.009G028200,GR.13			Thecc1EG037759(0.444)	GSVIVT01015282001(1.200)
	GrGLU41	Gorai.004G282300	Gorai.009G028200,Gorai.010G078300,GR.01				GSVIVT01015282001(1.595)
	GrGLU11	Gorai.009G028200	Gorai.004G282300,Gorai.010G078300,GR.01,GR.13	GR.03		Thecc1EG037759(0.457)	GSVIVT01015282001(1.197)
	GrGLU12	Gorai.009G028300			AT4G29360(1.877)		
					AT5G56590(1.796)		
	GrGLU28	Gorai.010G126400					
	GrGLU36	Gorai.004G094500					
	GrGLU16	Gorai.009G179400	GR.10,GR.11		AT1G11820(2.219)		
	GrGLU35	Gorai.004G064200					GSVIVT01030716001(1.136)
	GrGLU32	Gorai.001G204900					
	GrGLU3	Gorai.002G174100	Gorai.005G159100,GR.08				GSVIVT01011682001(1.440)
	GrGLU5	Gorai.005G159100	Gorai.002G174100,GR.12,GR.08				GSVIVT01011682001(0.962)
							GSVIVT01030716001(1.820)
**E**	GrGLU18	Gorai.009G242500	GR.05,GR.10,GR.09				
	GrGLU40	Gorai.004G257700	GR.03,GR.13				
	GrGLU13	Gorai.009G074100	GR.06				
	GrGLU42	Gorai.006G079400	GR.11,GR.06				
	GrGLU43	Gorai.006G079600					
	GrGLU21	Gorai.010G003500	GR.02,GR.05,GR.09,GR.09	GR.03,GR.05,GR.07,GR.08,GR.08			
	GrGLU20	Gorai.010G003400					
	GrGLU23	Gorai.010G003700					
	GrGLU22	Gorai.010G003600					
	GrGLU45	Gorai.006G134600					
	GrGLU46	Gorai.006G134700					
	GrGLU60	Gorai.008G159300	GR.05,GR.12,GR.09				
**F**	GrGLU57	Gorai.007G287700	Gorai.013G202800,GR.04,GR.13				

	GrGLU63	Gorai.013G202800	Gorai.007G287700,GR.13,GR.04				
	GrGLU59	Gorai.008G101400	GR.03				
	GrGLU51	Gorai.011G114400	GR.07				
	GrGLU53	Gorai.011G114600					
	GrGLU52	Gorai.011G114500					
	GrGLU25	Gorai.010G068800	GR.04,GR.01,GR.09	GR.09		Thecc1EG037932(0.444)	
	GrGLU27	Gorai.010G094500	GR.01				
	GrGLU64	Gorai.013G221700	GR.01,GR.10,GR.04,GR.09				
	GrGLU37	Gorai.004G097500					
	GrGLU8	Gorai.012G009200					
	GrGLU9	Gorai.012G043900					
	GrGLU66	Gorai.013G270800	GR.03,GR.09		AT5G58480(2.050)		
**G**	GrGLU67	Gorai.N004800	Gorai.001G230200,GR.07				
	GrGLU34	Gorai.001G230200	Gorai.N004800,GR.01,GR.07		AT3G07320(2.257)	Thecc1EG002746(0.461)	
	GrGLU4	Gorai.005G026800	Gorai.006G224700,GR.11		AT3G23770(2.098)	Thecc1EG020208(0.615)	
					AT4G14080(1.833)		
	GrGLU48	Gorai.006G224700	Gorai.005G026800,GR.11		AT3G23770(1.928)	Thecc1EG020208(0.536)	
					AT4G14080(1.965)		
**H**	GrGLU62	Gorai.013G019700	Gorai.011G279200,GR.12,GR.09,GR.10	GR.06,GR.07	AT3G55780(1.802)	Thecc1EG042611(0.551)	GSVIVT01016379001(1.511)
	GrGLU55	Gorai.011G279200	Gorai.013G019700,GR.10,GR.12			Thecc1EG042611(0.650)	GSVIVT01016379001(1.639)

* Ks values of gene pairs in syntenic blocks; and the chromosome No. indicated that homologous blocks can be found between two chromosomes, but the paralog genes was lost.

Supplementary Table 3. Expression clusters of β-1,3-glucanase genes in *G. hirsutum*.

Cluster Ⅰ	Cluster Ⅱ	Cluster Ⅲ	Cluster Ⅳ	Cluster Ⅴ	Cluster Ⅵ	Cluster Ⅶ	Cluster Ⅷ
GhGLU19D	GhGLU12D	GhGLU30A	GhGLU65A	GhGLU45A	GhGLU1A	GhGLU49A	GhGLU39D
GhGLU28D	GhGLU21D	GhGLU31A	GhGLU65D	GhGLU45D	GhGLU1D	GhGLU18A	GhGLU44A
GhGLU36A	GhGLU22D	GhGLU31D	GhGLU15A	GhGLU54bA	GhGLU39A	GhGLU18D	GhGLU40D
GhGLU36D	GhGLU46A	GhGLU21A	GhGLU50D	GhGLU54aD	GhGLU30D		
GhGLU10D	GhGLU46D		GhGLU50A	GhGLU51D	GhGLU19A		
GhGLU5D	GhGLU12A		GhGLU27D	GhGLU59D	GhGLU34A		
GhGLU5A	GhGLU42A		GhGLU61A	GhGLU53D	GhGLU56A		
GhGLU37A	GhGLU42D		GhGLU61D	GhGLU51A	GhGLU10A		
GhGLU37D	GhGLU43A		GhGLU17D	GhGLU54aA	GhGLU56D		
GhGLU2A	GhGLU43D		GhGLU17A	GhGLU53A	GhGLU34D		
GhGLU2D			GhGLU64A	GhGLU52D			
GhGLU33A			GhGLU64D				
GhGLU33D			GhGLU27A				
GhGLU35A			GhGLU57A				
GhGLU35D			GhGLU57D				
GhGLU16A							
GhGLU16D							
GhGLU41A							
GhGLU41D							
GhGLU26A							
GhGLU26D							
GhGLU28A							
GhGLU8A							
GhGLU8D							
GhGLU3A							
GhGLU3D							
GhGLU25D							
GhGLU9A							
GhGLU24A							
GhGLU32A							
GhGLU32D							
GhGLU66D							
GhGLU66A							
GhGLU24D							
GhGLU9D							
GhGLU63D							
GhGLU63A							
GhGLU67A							
38 Genes	10 Genes	4 Genes	15 Genes	11 Genes	10 Genes	3 Genes	3 Genes

Supplementary Table 4. Percentage of wilted leaves after *V. dahlia* inoculation.

Treatment	Days after *V. dahlia* inoculation					
	11	15	20	25	30	35
**JUN 1**	20.7±0.6 a	47.1±2.7 a	70.6±4.9 a	82.8±4.5 a	96.2±1.0 a	100±0.0 a
**TRV2:GLU43**	14.5±1.5 b	34.5±1.5 b	56.1±3.0 b	65.9±3.6 b	83.0±9.7 b	97.8±3.9 a
**TRV2:GLU42**	11.5±1.7 c	28.7±5.5 b	49.4±4.9 b	60.3±3.8 b	83.3±3.8 b	96.2±3.9 a
**TRV:00**	0.0±0.0 d	20.4±2.8 c	28.3±2.8 c	40.1±4.1 c	56.6±6.1 c	60.3±2.3 b
**CK**	0.0±0.0 d	10.1±3.1 d	25.1±1.2 c	36.0±2.5 c	50.5±3.8 c	57.1±1.2 b

The experiments were repeated three times. Each independent experiment with 20 cotton plants was performed and an average value of “percentage of wilted leaves” was calculated. Standard deviation reflects the differences among the three independent biological experiments. Different letters indicate the significant difference among different treatments (Tukey’s multiple comparison test, $P < 0.05$).

Supplementary Table 5. Oligonucleotide primers used in this study.

Gene	Forward (5'-3')	Reverse (5'-3')	Purpose
GLU33	ATTTCCGACGGGATACTGAAAGAG	CACAAAGGAAGTAAGTTGCTAACA	qRT-PCR expression analysis
GLU2	ACATGCCGGTATCGACAACAG	CATTAAACCCAAACTCTTTCC	qRT-PCR expression analysis
GLU41	GATCGCTGTTATCTTGAACCTG	GAAAATACATCCATGGCTTACATT	qRT-PCR expression analysis
GLU3	GCGCTTTCCTTATGACTGCT	CAATAGGAGAAACAAAATTGGGTT	qRT-PCR expression analysis
GLU35	GTTTCACTGGCATTTTAGTGTTGA	CCGGTTCCTCCATCTTTGTAC	qRT-PCR expression analysis
GLU26	TAAACCCCGAAAAGGTGTTGGAT	AGCAAGTTCTTCCCCACCGCAAAT	qRT-PCR expression analysis
GLU37	TTCTGTTCTTCTCCTCCTATTGC	TTCACAGTTTGCCTTATAGATAGC	qRT-PCR expression analysis
GLU16	TTGCATTGAATTCAAGATGATCTC	CGCATTCATTCAACGACTCA	qRT-PCR expression analysis
GLU28	CGATATGTAATCTCAGCCGCTTGT	GGCTTTGCTCGCGGATTTC	qRT-PCR expression analysis
GLU24	GGGCTTCATTCAGTTGATG	CCTAAAGATCCAAAATCAGGTATG	qRT-PCR expression analysis
GLU66	TATTGGGTTTCACATGGTAGAGA	AACTGAAAACCAGCAACTAACAGA	qRT-PCR expression analysis
GLU43	AGGCATTGGGATATTGCTTCTAAG	AAAAGACAAAACTCCCCAAATTCC	qRT-PCR expression analysis
GLU42	GAGTTAGAGAGACACTTTGGGTTG	AAAAAGACCAAATTTCCCAGGCTT	qRT-PCR expression analysis
GLU21	CAAACAGGAGAAATACCCAATTAG	CTGGAATTACAATAATCAGACCAC	qRT-PCR expression analysis
GLU22	GTTTTTCCCAAATAAACAGGAGAA	ATACCCGAGACTGAGAGAAATGGC	qRT-PCR expression analysis
GLU45	CCAAATAGGCAGCCTAAATACCAA	TTCCTAGGGAGAAGTGATACGCTC	qRT-PCR expression analysis
GLU59	CCATGTCCTGCAGATTCAATACTC	CCAAACTCATTTTATGGAATGGTG	qRT-PCR expression analysis
GLU17	GGAGTACATGAAAAAGGAGAGTAG	GCCAATCACTCAATACCATTAC	qRT-PCR expression analysis
GLU65	AACTATTCAGTGAATCAAGGGTCA	AAAAACCTAACTAGGCCACTTACA	qRT-PCR expression analysis
GLU63	CAAGGTTTGGCTATAATTACAACA	AGGGACTTACTGAAGTAAAAGAAG	qRT-PCR expression analysis
GLU30	ATCCAGTTGGTCTGGCTCCTACTC	AAATGCAAGTGCAGAACAGAATGA	qRT-PCR expression analysis
GLU9	AGGTGGGTTGTCGGCTGTA	GATACTATCAGCTTCATACGAGGG	qRT-PCR expression analysis
GLU18	CCAATATGAACCCTGTTTATCCA	TGCAGAAACTATATGAAGCGTCTC	qRT-PCR expression analysis
GLU19	TATCAGAAGAACACACGTGCG	TTTTCCCCTTTGTTTTTCCAC	qRT-PCR expression analysis
GLU1	CTGTCAATTTCCCACAGGATA	ATTACAACCCTCATCCTTGTC	qRT-PCR expression analysis
GLU34	ATTTTAACGGCTTGGCTACCCAGA	TATTCGATTCGTGATTGGGTATCC	qRT-PCR expression analysis
GLU42	CGCGGATCCATTCGATGCAATGTTGGATTC	CGAGCTCAGCAATATCCCAATGCCTTCC	VIGS analysis (underlined BamHI/SacI enzyme sites)
GLU43	CGCGGATCCTATATGGGATCAAGGACGTGG	CGAGCTCATTTTGGTTGCTTGTTTGGTG	VIGS analysis (underlined BamHI/SacI enzyme sites)
histone3	GAAGCCTCATCGATACCGTC	CTACCACTACCATCATGG	Reference gene for qRT-PCR analysis
